# Prevalence and associated factors of nomophobia among the medical and university students of Bangladesh

**DOI:** 10.1371/journal.pone.0341524

**Published:** 2026-08-31

**Authors:** Tonmoy Chowdhury, Saurya Dhungel, Mohammad Azmain Iktidar, Sreshtha Chowdhury, Purzia Tanaz Haque, Eshita Dey, Bikramaditya Chakma, Arefin Naher Oishee, Ishrat Jahan, Arpita Mazumder, Simanta Roy

**Affiliations:** 1 School of Research, Chattogram, Bangladesh; 2 Department of Epidemiology, University of Washignton School of Public Health, Seattle, Washington, United States of America; 3 Central Department of Public Health, Institute of Medicine, Tribhuvan University, Kathmandu, Nepal; 4 Directorate General of Health Services, Dhaka, Bangladesh; 5 Department of Public Health, North South University, Dhaka, Bangladesh; 6 Shaheed M Monsur Ali Medical College, Dhaka, Bangladesh; 7 Rangamati Medical College, Rangamati, Bangladesh; 8 Dinajpur Medical College, Dinajpur, Bangladesh; Chittagong Medical College, BANGLADESH

## Abstract

**Background:**

Nomophobia, characterised by the dread or anxiety of being without access to a mobile phone, has become an increasing behavioural issue among young adults, especially university and medical students. Excessive reliance on smartphones has been linked to psychological suffering, impaired everyday functioning, and negative academic results. Nonetheless, information about the prevalence and determinants of nomophobia among medical and university students in Bangladesh is scarce. This study aimed to evaluate the prevalence of nomophobia and investigate its associated sociodemographic traits and smartphone-related behaviors among medical and university students in Bangladesh.

**Materials and methods:**

A cross-sectional study was performed with 436 undergraduate medical and non-medical students from eight districts of Bangladesh from September 2023 to July 2025 to encompass diversity throughout academic cycles and guarantee wider geographic representation, using an online structured questionnaire. Participants were recruited using non-probability snowball sampling through peer networks and institutional connections. Nomophobia was evaluated via the validated Nomophobia Questionnaire (NMP-Q). The questionnaire was pilot-tested (n = 60) and showed good reliability (Cronbach’s α = 0.82). Information on socio-demographics, mobile phone usage trends, and application preferences was collected. Data was processed using STATA version 16. Descriptive statistics, t-tests, ANOVA, Pearson correlation, and multiple linear regression analyses were used to ascertain factors related to nomophobia. Effect sizes were reported as β coefficients with 95% confidence intervals. Reporting of effect sizes alongside p-values allows better interpretation of the practical significance of findings beyond statistical significance.

**Results:**

The average age of participants was 20.70 ± 1.52 years. 46.79% of students exhibited moderate nomophobia, whereas 25.69% had severe nomophobia. Elevated nomophobia scores were significantly associated with female gender, moderate household income, usage of social media and communication applications, prolonged daily mobile phone usage (>7 hours), frequent phone checking, and instant phone checking upon awakening (effect sizes from adjusted models: > 7 hours usage β = 13.20; 95% CI: 1.31–25.10; social communication application use β = 8.25; 95% CI: 2.63–13.86; immediate phone checking β = 7.09; 95% CI: 0.67–13.52). Multiple linear regression indicated that extended phone usage, engagement with social communication applications, middle-income position, and early-morning phone checking were associated with elevated nomophobia scores.

**Conclusion:**

Nomophobia is significantly common among medical and university students in Bangladesh. Behavioural patterns such as prolonged daily smartphone use, frequent phone checking, immediate phone use upon waking and extensive engagement with social communication and media applications are associated with excessive smartphone usage and the development of nomophobia; however, causality cannot be inferred due to the cross-sectional study design. These findings underscore the necessity for awareness initiatives, early detection, and focused interventions to alleviate the adverse psychological and behavioural effects of nomophobia in students within the socio-cultural and digital context of Bangladesh, where rapid smartphone penetration may contribute to these patterns.

## Introduction

Nomophobia- short for ‘No Mobile Phone Phobia’ – refers to discomfort, apprehension, nervousness, or distress attributable to the inability to access a mobile phone [1,2] and is progressively framed within the extensive context of behavioral addiction and problematic smartphone usage in modern psychiatric and public health literature [3]. In 2008, UK-based research institute introduced the term ‘Nomophobia’. They conducted a study which found that 53% of cell phone users exhibit anguish when they are faced with troubles regarding their phone [4]. Recent global research indicates significantly elevated prevalence estimates, especially among young adults [5]**.** Investigations reveal that Nomophobia is an emerging concern in the present times [6].

Mobile phones, an essential feature of our daily lives [7] have evolved into a multidimensional high-efficiency device that can be used as a media player, a tool for social networking, for playing video games and music, as a digital camera, for browsing [8] calls, texts, paying bills, and carrying out online transactions, among other functions [9]. This wide range of usage of phones makes it increasingly difficult to identify disorders like nomophobia as functional utility overlaps with maladaptive use patterns, complicating clear clinical delineation [3]. Globally, the rate of subscription to cell phones is 103.5 per 100 population [10]. According to World Bank Trend statistics, in 2023, Bangladesh recorded over 114 mobile cellular subscriptions per 100 individuals, indicating an average of over one SIM card per person, attributable to multiple SIM ownership [11]. Published population health research indicates that subscription density rose from 0.22 per 100 individuals in 2000 to 107 by 2020 in Bangladesh [12]. The swift digital growth positions Bangladesh in a high-risk context for smartphone-related behavioral issues, while subscription density does not immediately correlate with individual usage intensity [13]. Mobile phones create a perception of social inclusion among users [14].

Different studies done in India have found that several demographic variables, such as gender, age, frequency of use, and level of education, show significant relations to Nomophobia. Specifically, women are more prone to Nomophobia than men, which is consistent with other studies that report a higher prevalence of Nomophobia among women [15–19] despite the heterogeneity of findings between regions, this indicates the presence of socio-cultural moderation effects [5]. Findings on gender differences in smartphone-related behaviors are inconsistent across studies [19]. While some research suggests that overall smartphone use may not differ substantially by gender, other university-based studies have reported differences in nomophobia scores between male and female students [20]. These findings may be substantiated since women frequently fear losing social connections and want to evade loneliness in public settings [21] aligning with theoretical constructs of social connectivity dependence and fear of missing out (FoMO) [22]. The age group of 18–25 is reportedly susceptible to Nomophobia [16,23]. Still, the duration of using a mobile phone happens to be the strongest predictor of nomophobia, especially those who spend more than three hours on their device per day are susceptible to severe nomophobia [16,18,23]. Another study found a correlation between nomophobia score and anxiety and depression [1,17,24,25] affirming its conceptual association with mental health outcomes and underscoring its significance as a public health issue [3].

The findings of another study revealed the prevalence of nomophobia among medical college students and interns in Southern Haryana, India, was nearly 40% [26]. The study also reported significant association of nomophobia with sleep quality and academic activities. Other studies have shown that prevalence of moderate or severe nomophobia ranges from 50% to 85% [27–30] although estimates varied significantly owing to variations in measurement instruments, populations, and research methodologies, this underscores the necessity for context-specific evidence [5]. Despite the increasing body of global and regional literature, there is a paucity of nationally representative information from Bangladesh, especially in contrasting medical and non-medical student populations and investigating behavioral correlations within a cohesive analytical framework [13]. Given the rising concern of nomophobia, this study aimed to evaluate the prevalence of nomophobia and investigate its associated sociodemographic traits and smartphone-related behaviors among medical and university students in Bangladesh.

## Methodology

### Study design and sampling technique

A cross-sectional study was carried out among undergraduate students from eight divisions in Bangladesh - Dhaka, Chattogram, Rajshahi, Khulna, Sylhet, Barisal, Mymensingh, and Rangpur. Participants were recruited through snowball sampling.

### Sample size

The sample size was calculated using the formula: n = z^2^ × p × (1 − p)/d^2^, where: z = 1.96 for a confidence level of 95%, p = proportion = 56% (from a previous study [31]), d = margin of error = 0.05, which yielded an initial sample size of 379. Considering the 15% non-response, the final sample size was, 379 + 56.9 = 435.9 ≈ 436. We approached 500 students to participate, among whom 436 (~87.2%) completed the survey and were included in the analysis. Eleven participants had incomplete surveys, and they were left out of the analysis.

### Study instrument and participants

The data was collected from the study participants using a structured online questionnaire between September 2023 to July 2025. The prolonged data collection period was deliberately structured to encompass heterogeneity across academic cycles, institutional calendars, and examination phases, thus enhancing the diversity and stability of responses despite the cross-sectional design. The duration of 22 months can be attributed to the snowball nature of the sampling, resource constraints and also our effort to recruit participants from all the eight divisions across the country. Since the study’s major goal was to examine the determinants and prevalence of nomophobia, it included only adult (over 18 years of age) students and owned any type of personal mobile phone, including a button phone, multimedia/feature phone, or smartphone or type of digital device used (Android, iPhone, Desktop, laptop, tablet, etc.). Students who were non-citizen of Bangladesh or did not own any type of personal mobile phone or digital device used (Android, iPhone, Desktop, laptop, tablet, etc.) were excluded from the study. Nomophobia was assessed using the 20-item Nomophobia Questionnaire (NMP-Q) [32]. The NMP-Q has been widely used internationally and a Bangla version has previously been psychometrically evaluated among Bangladeshi university students [33]. In the present study, the questionnaire was pilot-tested among 60 students before data collection to assess clarity, acceptability, and internal consistency. The pilot test showed good internal consistency reliability, with a Cronbach’s alpha (α = 0.82).

### Independent variables

Information about the respondent’s socioeconomic status (age, sex, occupation, and place of employment), type of digital device used (Android, iPhone, Desktop, laptop, tablet, etc.), type of application or social media used (Facebook, WhatsApp, or any streaming media), and self-reported average duration of daily device use (2 hours, 2–4 hours, 5–7 hours, or >7 hours), use of phone before going to sleep and soon after waking up were assessed. All participants who provided their consent to be included before their data were collected.

### Dependent variables

The 20-item NMP-Q questionnaire [32] we used for measuring nomophobia is based on a seven-point Likert scale, where 1 indicates ‘does not agree at all’ and 7 indicates ‘strongly agree’. Higher scores indicate higher levels of nomophobia. A study conducted in adolescents and young university adults also used the Nomophobia Questionnaire (NMP-Q) instrument and the standard cut-off points [20], where a score of 20 indicates absence; scores between 21 and 59 indicates mild level; scores between 60 and 99 indicate moderate level; and scores of 100 or above indicate severe level [34]. Furthermore, 1–60, 61–100, 101–140 indicated mild, moderate, and severe nomophobia, respectively [7,34]. In the context of inconsistencies in earlier reported NMP-Q cut-off classifications, this study followed the widely recognized classification (20 = absence; 21–59 = mild; 60–99 = moderate; ≥ 100 = severe) [32,34,35] for all analyses to maintain consistency and comparability, while also acknowledging alternative classifications.

### Statistical analysis

STATA, version 16, was used to analyze the data. The quantitative data were summarized using the mean and the standard deviation. The frequency of occurrence of a categorical variable was stated as a percentage. We used the t-test and ANOVA to look at the association between categorical variables with mean NMP-Q scores, and multiple linear regression to identify factors independently associated with the continuous NMP-Q total score. The variables were selected from extensive literature review and through bivariate analysis. Selection of covariates for the multiple regression model was guided mainly by theoretical relevance and evidence from previous literature, including gender, average daily phone usage, use of social networking/communication applications, and checking the phone immediately after waking. Variables without strong prior theoretical support were included only if they demonstrated statistically significant associations with NMP-Q scores in bivariate analysis. Accordingly, household income was retained in the final model because it was significantly associated with nomophobia in the bivariate analysis at p < 0.05. Regression results were reported as unstandardized β coefficients with corresponding 95% confidence intervals and p-values. The variance inflation factor (VIF) was used to assess multicollinearity. Regression model assumptions, including normality of residuals, linearity, and homoscedasticity, were evaluated using residual diagnostics. For other parametric analyses, normality was evaluated using graphical methods and the Shapiro–Wilk test, while homogeneity of variance was assessed using Levene’s test. No major violations were detected. Statistical significance was set at α < 0.05. Missing data were minimal (<5%) and were handled using complete-case analysis under the assumption of missing at random.

### Ethical consideration

The Institutional Review Board of Public Health Foundation Bangladesh approved the research (Approval no: PHFBD-ERC-IP09/2023), and all participants provided informed written consent. Wherever feasible, the 1964 Declaration of Helsinki and later modifications, latest on October 2024 and comparable ethical standards were followed. Data collection was voluntary, and no incentives were offered to participants. Data was only accessible to the research team and were not disclosed anywhere.

## Results

The study involved 436 participants, with a mean age of (20.70 ± 1.52) years and a mean BMI of (22 ± 3.88) kg/m^2^ (Table 1). The majority of the participants were enrolled in medical school (53.4%), female (53%), and from Chattogram (46.3%). Additionally, a significant portion of the participants (54.2%) lived away from their families. Most participants (57.3%) came from well-off families whose monthly income was 30000–60000 Bangladeshi Taka (BDT) [36,37]. Among the participants, android smartphones were the most commonly used device (92.7%), while a smaller proportion used laptops (30.7%). Other devices used included button phones (6.2%), iPhones (9.9%), desktops (6.4%), and tablets (4.8%). The majority of participants accessed the internet via Wireless Fidelity **(**Wi-Fi) (75.5%), while a smaller portion used mobile/cellular data (22.7%). Social media applications (e.g., Facebook, Instagram) were predominantly used by participants (73.6%), followed by social communication applications (e.g., WhatsApp, Messenger; 67.9%). A notable proportion (49.3%) indicated an inclination towards using music and video sharing applications (e.g., YouTube, Netflix, Spotify, TikTok). A similar picture was portrayed when the purpose of their mobile phone use was asked. The majority of the participants (82.6%) used it to communicate with family and friends. Almost equal numbers of participants used their mobile phones for browsing and sharing on social media, and to follow the news and current events. The majority (41.5%) of all participants used their phones daily for 2–4 hours. A large majority (94.5%) used their phones before going to bed, and upon waking up, most of them (78.4%) immediately checked their devices (Table 1).

**Table 1:** Background Characteristics of Study Participants (n = 436).

Variable	Frequency	Percentage
Age (in year), (mean±SD)	20.70 ± 1.52	
BMI (in kg/m2), (mean±SD)	22 ± 3.88	
**Type of student**		
Medical Student	233	53.4
Non-medical Student	203	46.6
**Gender**		
Male	205	47.0
Female	231	53.0
**Residence**		
Home with Family	196	45.8
Home without family	232	54.2
**Marital Status**		
Unmarried	431	98.9
Married	4	0.9
Divorced/Separated	1	0.2
**Academic Year**		
1st	232	53.2
2nd	128	29.36
3rd	19	4.4
4th	26	6.0
5th	31	7.1
**Division**		
Dhaka	119	27.3
Chattogram	202	46.3
Rajshahi	21	4.8
Khulna	17	3.9
Barisal	9	2.1
Sylhet	14	3.2
Mymensingh	8	1.8
Rangpur	46	10.6
**Income (in BDT)**		
<15,000	53	12.2
15,000-30,000	133	30.5
30,001-60,000	250	57.3
**Types of Device Use**		
Android Smart Phone	404	92.7
Button Phone	27	6.2
iPhone	43	9.9
Laptop	134	30.7
Desktop	28	6.4
Tablet	21	4.8
**How do you use Internet**		
Via Wi-Fi	329	75.5
Via Mobile Data	99	22.7
Both Mobile Data and Wi-Fi	8	1.8
**Most commonly used application** ^**a**^		
Social media applications (Facebook, Instagram etc.)	321	73.6
Social network/communication application (WhatsApp, Messenger etc.)	296	67.9
Games applications	74	17.0
Music, Video sharing applications (YouTube, Netflix, Spotify, TikTok, etc.)	215	49.3
Mobile Banking Application (Bkash, Nagad etc.)	92	21.1
Shopping Application (Daraz etc.)	40	9.2
**Average phone usage time per day**		
Less than 2 h	31	7.1
2-4 h	181	41.5
5-7 h	154	35.3
More than 7 h	70	16.1
**Looking at phone as soon as waking up**	342	78.4
**Use phone before going to bed**	412	94.5
**Frequency of checking mobile phone**		
Each 5 minutes	45	10.3
Each 10 minutes	77	17.7
Each 20 minutes	72	16.5
Each 30 minutes	100	22.9
Each hour	142	32.6
**The purpose of mobile phone use** ^**a**^		
Listen to Music	208	47.7
Play games	89	20.4
Browse/ share on social media	226	51.8
To obtain different information	210	48.2
To follow the news and current events	225	51.6
Sharing own thoughts	115	26.4
Communicating with friends, family and relatives	360	82.6

^a^Multiple Response.

BDT, Bangladeshi Taka; BMI, body mass index; SD, standard deviation.

**Fig 1:**
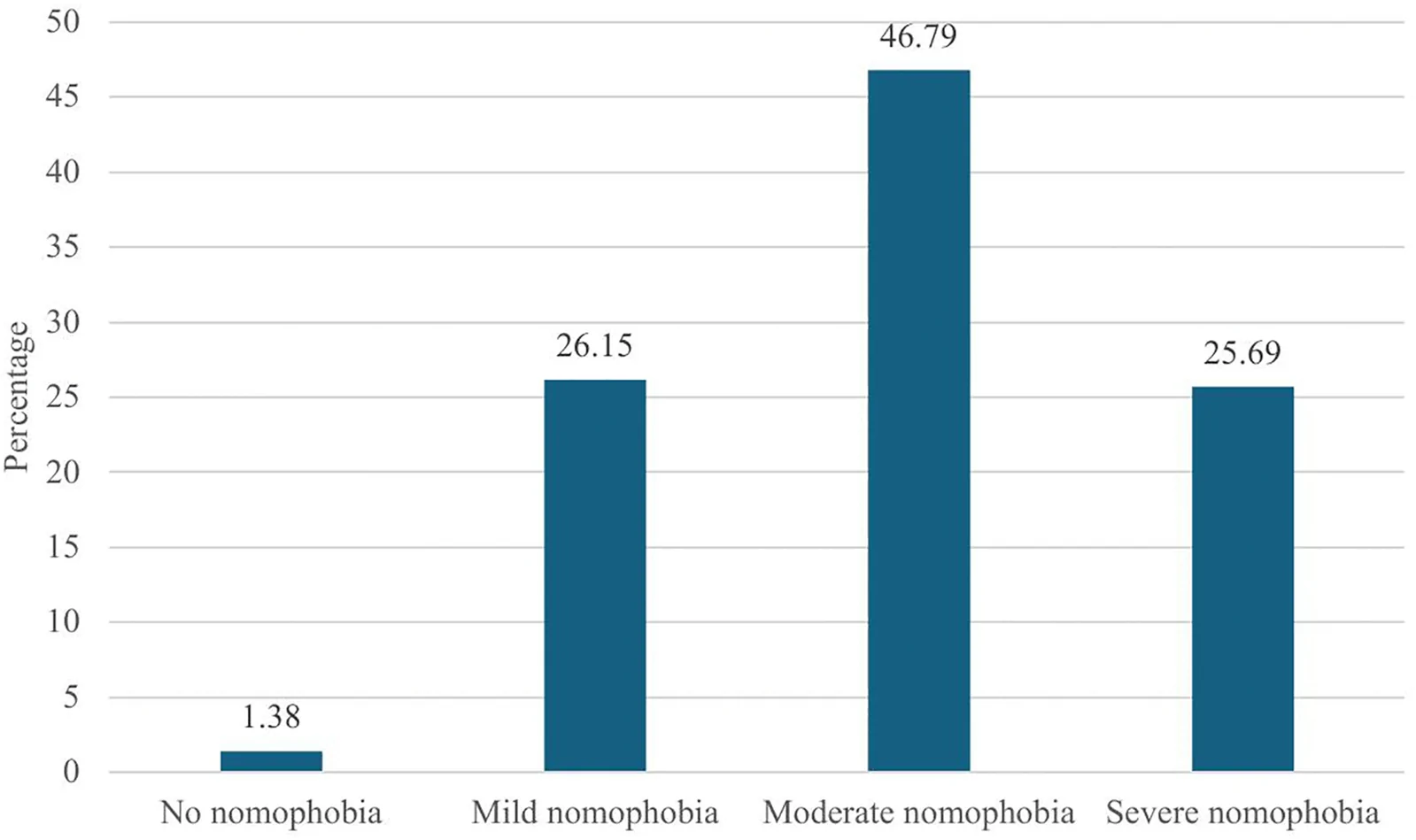
Prevalence of nomophobia among the study participants.

The Fig 1 classifies nomophobia severity according to the Nomophobia Questionnaire (NMP-Q). The percentages of participants in each category are shown above the respective bars in Fig 1. The majority of participants exhibited moderate nomophobia (46.79%), followed by mild (26.15%) and severe nomophobia (25.69%), while only 1.38% had no nomophobia, which has been described in Fig 1 (Fig 1: Prevalence of nomophobia among the study participants).

Among the students, factors such as gender (p-value = 0.018), income in BDT (p-value = 0.044), social media applications use (p-value = 0.014), social communication application use (p-value = 0.005), music, video sharing application use (p-value = 0.002), mobile banking application use (p-value = 0.036) and shopping application use (p- value = 0.040) had significant relation with having higher nomophobia score (Table 2). Average phone usage per day (p-value = 0.022), looking at phone as soon as waking up (p-value = 0.010) and frequency of checking mobile phone (p-value = 0.006) had a significant association with developing nomophobia (Table 2). To maintain clarity and readability, key findings are summarized in the text while detailed distributions are presented in tables, minimizing redundancy between textual and tabular data. Effect sizes (Table 2) were examined to assess the magnitude of associations in addition to statistical significance. In the bivariate analysis, most statistically significant associations showed small effect sizes. The difference in NMP-Q scores between medical and non-medical students was negligible (Cohen’s d = 0.06), while gender showed a small effect size (Cohen’s d = 0.23). Small effect sizes were also observed for income level (η² = 0.014), use of social media applications (η² = 0.014), use of social network/communication applications (η² = 0.018), use of music/video-sharing applications (η² = 0.021), average phone usage per day (η² = 0.021), checking the phone immediately after waking (η² = 0.015), and frequency of checking the mobile phone (η² = 0.033). These findings indicate that, although several variables were statistically associated with NMP-Q scores, their magnitude were generally small.

**Table 2:** Bivariate relationship between the participants’ characteristics and NMP-Q total score (n = 436).

Variable	Mean	SD	p-value	Effect Size
**Type of Student**			0.520^a^	d = 0.06
Medical	78.1	27.6		
Non-medical	79.9	29.2		
**Gender**			**0.018** ^a^	**d = 0.23**
Male	82.3	28.9		
Female	75.9	27.5		
**Division**			0.937^b^	η²=0.005
Dhaka	76.5	29.8		
Chattogram	79.0	26.8		
Rajshahi	82.7	29.3		
Khulna	79.6	32.8		
Barisal	78.4	27.2		
Sylhet	82.6	22.6		
Mymensingh	75.5	24.9		
Rangpur	82.6	32.1		
**Income (in BDT)**			**0.044** ^b^	**η²=0.014**
<15,000	72.2	32.7		
15,000-30,000	83.2	28.5		
30,001-60,000	78.1	27.0		
**Residence**			0.567^a^	η²=0.001
Home with family	78.2	28.9		
Home without family	79.7	27.6		
**Marital Status**			0.148^a^	η²=0.009
Unmarried	78.9	28.2		
Married	71.0	34.2		
Divorced/	132.0	0.0		
**Academic Year**			0.176^b^	η²=0.015
1st	78.8	28.1		
2nd	75.9	28.4		
3rd	76.7	23.4		
4th	88.8	31.0		
5th	85.5	29.6		
**Device Usage**			t-test	
Android Smart Phone	79.1	27.7	0.701^c^	η²=0.001
Button Phone	83.7	33.3	0.362 ^c^	η²=0.002
iPhone	81.7	33.2	0.492 ^c^	η²=0.001
Laptop	82.1	28.5	0.123 ^c^	η²=0.005
Desktop	84.7	29.7	0.264 ^c^	η²=0.003
Tablet	90.6	29.9	0.053 ^c^	η²=0.009
**Internet Use**			(t-test)	
Via Wi-Fi	79.1	27.7	0.768 ^c^	η²=0.002
Via Mobile Data	78.8	30.7	0.965 ^c^	η²=0.001
Both Mobile Data and Wi-Fi	70.9	24.8	0.418 ^c^	η²=0.001
**Application Used**			(t-test)	
Social media applications (Facebook, Instagram etc.)	80.9	29.0	**0.014** ^c^	**η²=0.014**
Social network/communication application (WhatsApp, Messenger etc.)	81.5	26.8	**0.005** ^c^	**η²=0.018**
Games applications	81.4	29.3	0.403 ^c^	η²=0.002
Music, Video sharing applications (YouTube, Netflix, Spotify, Tiktok, etc.)	83.1	27.3	**0.002** ^c^	**η²=0.021**
Mobile Banking Application (Bkash, Nagad etc.)	84.4	28.2	**0.036** ^c^	**η²=0.010**
Shopping Application (Daraz etc.)	87.7	29.5	**0.040** ^c^	**η²=0.009**
**Average phone usage per day**			**0.022** ^**b**^	**η²=0.021**
Less than 2 h	67.9	28.2		
2-4 h	77.3	27.0		
5-7 h	79.9	26.9		
More than 7 h	85.8	33.1		
**Looking at phone as soon as waking up**			**0.010** ^**a**^	**η²=0.015**
Yes	80.7	29.1		
No	72.3	24.3		
**Using phone before going to bed**			0.093^a^	η²=0.007
Yes	79.5	28.3		
No	69.5	28.6		
**Frequency of checking mobile phone**			**0.006** ^**b**^	**η²=0.033**
Each 5 min	80.1	36.7		
Each 10 min	80.5	29.7		
Each 20 min	82.6	27.4		
Each 30 min	84.6	26.0		
Each hour	71.8	25.4		
**The purpose of mobile phone use**				
Listen to Music	81.9	27.2	**0.036** ^c^	**η²=0.010**
Play Games	78.3	29.5	0.818 ^c^	η²=0.001
Browse/share on social media	82.1	27.4	**0.015** ^c^	**η²=0.013**
To obtain different information	82.0	27.0	**0.026** ^c^	**η²=0.011**
To follow the news and current events	84.2	26.6	**<0.001** ^c^	**η²=0.036**
Sharing own thoughts	83.9	29.6	**0.027** ^c^	**η²=0.011**
Communicating with friends, family and relatives	79.5	27.9	0.369 ^c^	η²=0.002

^a^p-values are determined from t-test.

^b^p-values are determined from ANOVA.

^c^p-values are determined from t-test with comparison to negative responses for each variable.

Effect sizes were interpreted using conventional thresholds [38,39]: Cohen’s d values of approximately 0.20, 0.50, and 0.80 were considered small, moderate, and large, respectively; η² values of approximately 0.01, 0.06, and 0.14 were considered small, moderate, and large, respectively.

**Significant (<0.05) p-values are in bold.**

We also calculated the Pearson correlation coefficient to assess the relation between nomophobia scores with age and body mass index (BMI) [data not provided in table]. Age had a statistically significant, although a weak positive relation with nomophobia scores (r = 0.12; p-value = 0.015). Moreover, BMI did not have any significant correlation with nomophobia scores (r = 0.04; p-value = 0.44).

In addition to p-values, effect sizes from multiple linear regression analyses are reported as β coefficients with corresponding 95% confidence intervals (Table 3), providing a more informative estimate of the magnitude and direction of associations between NMP-Q scores and the independent variables. The estimates shown in Table 3 were derived from a multiple linear regression model after simultaneous adjustment for gender, income, usage of social networking applications, average daily phone use, and checking the phone immediately upon waking.

**Table 3:** Multiple linear regression for the predictors of NMP-Q total score.

Variables	β	95% CI	p-value
**Gender**			
Male	Ref.		
Female	−5.24	(−10.57, 0.10)	0.054
**Income (in BDT)**			
<15,000	Ref.		
15,000-30,000	11.48	(2.57, 20.39)	**0.012**
30,001-60,000	6.74	(−1.58, 15.07)	0.112
**Use of social network/communication application (i.e., WhatsApp, Messenger etc.) use**			
No	Ref.		
Yes	8.25	(2.63, 13.86)	**0.004**
**Average phone usage per day**			
Less than 2 h	Ref.		
2-4 h	4.71	(−6.01, 15.42)	0.388
5-7 h	5.66	(−5.37, 16.69)	0.314
More than 7 h	13.20	(1.31, 25.10)	**0.030**
**Looking at phone as soon as waking up**			
No	Ref.		
Yes	7.09	(0.67, 13.52)	**0.031**

*Note: R^2^: 0.0725.

Adjusted R^2^: 0.0618.

After adjusting for demographic and other variables (gender, income, etc.) participants who had an income between 15,000–30,000 BDT had on average, 11.48 units higher nomophobia scores (β = 11.48; 95% CI: 2.57, 20.39; p = 0.012) than those who earned less than 15,000 BDT (Table 3). Furthermore, people using social media had on average 8.25 units higher nomophobia scores (β = 8.25; 95% CI: 2.63, 13.86; p = 0.004) than those who were not using any social media platforms, after adjusting for demographic and other variables (gender, income, etc.). Participants using mobile phones for more than 7 hours had on average, 13.20 units higher nomophobia scores (β = 13.20; 95% CI: 1.31, 25.10; p = 0.030) than those who used mobile phone for less than 2 hours, after adjusting for demographic and other variables (gender, income, etc.). Additionally, participants who looked at their mobile phones as soon as they woke up had on average, 7.09 units higher nomophobia scores (β = 7.09; 95% CI: 0.67, 13.52; p = 0.031) than those who did not, after adjusting for demographic and other variables (gender, income, etc.). Regression model assumptions for normality and homoscedascity of residuals were checked, and no significant violations were identified, supporting the validity of the reported estimates.

## Discussion

The study revealed a significant prevalence of nomophobia among university students in Bangladesh, with approximately 73% exhibiting moderate to severe symptoms, highlighting a considerable behavioral issue. Female students and those from middle-income households exhibited markedly elevated nomophobia ratings, underscoring the impact of demographic and socioeconomic variables. Extended daily mobile phone usage (>7 hours), and instant checking of phone upon awakening were significantly correlated with elevated nomophobia levels. Usage of social media and communication platforms was substantially associated with elevated nomophobia scores. While the findings are consistent with prior studies [5], they may also be interpreted through behavioral dependence and cognitive salience frameworks [3], where excessive smartphone use reinforces habitual checking behavior and anxiety related to disconnection [3,22,30]. This theoretical perspective elucidates the connection between higher usage patterns and the rise of nomophobia, extending beyond mere descriptive associations [3,40].

In our study 53.4% of the total participants were medical students and most of the participants were more addicted to mobile phones. A similar cross-sectional study conducted in India concluded that all medical students had various degrees of nomophobia which is really concerning [41]. A cross-sectional study was carried out in March, 2021 in Pakistan which led to the conclusion that nomophobia, in mild to severe forms, affects the majority of undergraduate students in Pakistan [42]. Our study also depicted that among the participants, 26.15% were mildly nomophobic, 46.79% were moderately nomophobic and 25.69% were severely nomophobic. Furthermore, a study done in November 2019 in India shows that a large fraction of the students displayed severe nomophobia symptoms, distinct usage habits, and false beliefs about their health and usage pattern. In a recent study done on Bangladeshi young adults, 61.4% had higher Problematic Use of Mobile Phone (PUMP) scores [13,43,44]. The Problematic Use of Mobile Phone (PUMP) scale is a validated self-reported tool designed to evaluate maladaptive, excessive, and dependency-like behaviors associated with mobile phone usage and their resultant effects [45]. Other studies in different countries conducted with NMP-Q showed results ranging from 42.6% to 67.2% with moderate or severe nomophobia [17,34,46,47]. Although academic specialization was considered as a potential determinant, it did not show a significant independent association in the adjusted analysis. This suggests that nomophobia may be influenced more by individual behavioral patterns and smartphone usage characteristics than by academic discipline alone.

In our study, we found a positive correlation between different ages and nomophobia. Similar findings were present in a study conducted in North India [17]. On the other hand, the relationship between age and nomophobia is inconsistent, with studies from Saudi Arabia and Turkey reporting no significant relationship between age and nomophobia [34,43]. In terms of gender, some studies have reported higher nomophobia in males, especially in adolescent boys, due to greater engagement in gaming, entertainment-related smartphone use, and other problematic digital behaviors. However, our study results indicated that female students were more vulnerable to nomophobia, which is consistent with the Turkish study that reported significant association of female gender with nomophobia [48]. One possible reason is that female students are more likely to use smartphones for social networking, emotional communication, reassurance and maintenance of interpersonal relationships. Previous research has shown that women are generally more likely than men to use mediated communication platforms (text messaging, social networking sites and video calls) [49]. Such greater dependence on smartphones for relational and emotional communication may expose them to higher levels of anxiety when disconnected from the device [50]. Furthermore, nomophobia is highly associated with fear of missing out, fear of losing communication access and psychological distress [3,22], while anxiety disorders are consistently found to be more common in females than males [50,51]. Thus, the gender difference found might not only be due to biological or demographic differences but also affected by variation in smartphone-use motives, social connectedness, emotional communication styles and vulnerability to anxiety. Females are often reported to use mediated communication platforms more for interpersonal and emotional communication, and to have higher anxiety-related vulnerability in relation to problematic smartphone use [3,49,51,52]. However, due to the mixed findings across studies, gender differences in nomophobia should be interpreted with caution and in the sociocultural and behavioral context of the study population [30,32,42,47].

Our study reveals the multi-purpose usage of mobile phones by students. Social networking, mobile banking, shopping and video sharing are the significant purposes of using phones that have association with nomophobia, similar to other studies [19,53]. In our study, there is a statistically significant relationship between the number of hours per day spent using a mobile phone and the severity of nomophobia. Those who use more than 7 hours of mobile phone had higher nomophobia scores. A similar association between prolonged daily phone use and nomophobia was exhibited in other studies [17,43]. Another study observed 62.1% of students spent time on smartphone for more than 3 hours [17]. In our study, 78.4% of students practice the habit of checking their phones as soon as they wake up, which has a strong correlation with the development of nomophobia. A similar association between instant checking of phone after waking up was observed by Hoşgör [54]. A similar association between nomophobia and frequency of checking mobile phones was observed by Khilnani [55]. According to a study conducted in Peru, nomophobia is a common and rising issue among college students, which manifests earlier in life and is linked to symptoms of anxiety or depression [24]. The mental health of the students might benefit from the use of evaluation and early intervention measures [24]. The variations observed by age and gender must be interpreted with care and integrated into a wider behavioral framework. Individuals who are younger might show increased nomophobia as a result of deeper digital engagement and reliance on social connections, while variations between genders could indicate sociocultural trends in communication styles and technology utilization instead of fundamental behavioral differences. These patterns align with the emerging global literature, yet caution should be exercised to avoid overgeneralization [5,31,32,49]. Instead of simply conforming to previous research, this study adds to the expanding evidence from low- and middle-income countries, emphasizing how sociocultural and technological changes in Bangladesh could create distinct patterns of smartphone dependency in contrast to high-income environments. These findings suggest that nomophobia is not merely a function of device availability but reflects a complex interaction between behavioral habits, psychological dependence, and environmental exposure.

This study possesses certain limitations that must be acknowledged when evaluating the results. The cross-sectional design limits the capacity to determine causal correlations between nomophobia and its related factors. The detected linkages indicate correlations at a specific moment and cannot ascertain directionality or temporal succession. The study utilised self-reported data obtained via an online questionnaire, which may be influenced by recall bias and social desirability bias. Participants may have inaccurately reported their mobile phone usage patterns and nomophobia-related behaviors, potentially affecting the accuracy of reported smartphone usage patterns and behavioral responses. Third, the implementation of snowball sampling and online data collecting may have resulted in selection bias, as students with consistent internet access and greater engagement with mobile devices were more inclined to participate. Additionally, participants are more likely to recruit individuals within similar social and academic networks. Thus, the sample may not comprehensively reflect all university students in Bangladesh, especially those from rural regions or lower socioeconomic strata. Fourth, significant psychological and behavioural confounders, including anxiety, depression, stress levels, sleep quality, and academic pressure, were not directly evaluated, despite existing evidence indicating correlations between them and nomophobia. The lack of information in these variables may have resulted in residual confounding in the regression analysis. Ultimately, although the NMP-Q is a validated and extensively utilised tool, it assesses perceived nomophobia rather than clinically recognised behavioral addiction. Moreover, variables such as social media/communication application use and daily duration of smartphone use may serve as potential mediators in the relationship between sociodemographic characteristics and nomophobia, but mediation analysis was not performed due to the limitations of the cross-sectional design, which does not allow for temporal inference and the clear establishment of the exposure-mediator-outcome sequence. Consequently, the results should be understood as indicative of nomophobia symptomatology rather than clinical illness. The categorization of academic specialization into broad groups (medical vs non-medical) may have limited the ability to detect more nuanced differences across specific disciplines. Although the Bangla version of the NMP-Q has previously been psychometrically validated among Bangladeshi university students, construct validity was not independently assessed in the current study sample using exploratory or confirmatory factor analysis [33]. However, NMP-Q is a widely used instrument and the pilot test for this study demonstrated good internal consistency and supported the clarity of the questionnaire. Therefore, although the pilot test supported the reliability and clarity of the tool. The absence of sample-specific psychometric validation should be considered when interpreting the findings. Despite the limitations, this study is important because understanding the prevalence and associated factors of nomophobia among mobile phone users can help in developing strategies to prevent its negative effects, such as discomfort, anger, anxiety, and feelings of insecurity [28]. While the study title emphasizes differences across academic disciplines, the findings indicate that such differences may not be pronounced when controlling for other behavioral and sociodemographic factors.

## Conclusion

In a nutshell, nomophobia is a pressing issue in today’s world. In this modern era, avoiding smartphones and devices is nearly impossible as we are hugely dependent on these devices in our day-to-day activities. Therefore, it is crucial to raise awareness of the negative impacts of nomophobia and conduct extensive research on it. This study highlights the need for targeted preventive and behavioral interventions. Preventive strategies such as digital literacy programs, behavioral awareness campaigns, and structured guidelines for healthy smartphone use among students should be considered. Institutional policies promoting balanced technology use and mental well-being may further help mitigate the growing burden of nomophobia. Future research should employ longitudinal designs to improve causal inference of findings.

## Supporting information

S1 File Dataset. (XLSX)
