# Supplementary material for: Prevalence and associated factors of nomophobia among the medical and university students of Bangladesh
Source: PLoS One. 2026 Aug 31;21(8):e0341524. doi: 10.1371/journal.pone.0341524 (PMC13528988; doi:10.1371/journal.pone.0341524)
Supplement: Supplementary file 1 [file pone.0341524.s003.docx]

**Response to Reviewer’s Comments**

**Journal Requirements:**

1. Please ensure that your manuscript meets PLOS ONE's style requirements, including those for file naming. The PLOS ONE style templates can be found at

<https://journals.plos.org/plosone/s/file?id=wjVg/PLOSOne_formatting_sample_main_body.pdf> and

[https://journals.plos.org/plosone/s/file?id=ba62/PLOSOne_formatting_sample_title_authors_affiliations.pdf](https://track.editorialmanager.com/CL0/https:%2F%2Fjournals.plos.org%2Fplosone%2Fs%2Ffile%3Fid=ba62%2FPLOSOne_formatting_sample_title_authors_affiliations.pdf/1/010f0199c1e53be4-edb81237-fa68-41c3-ba79-2c285ef02365-000000/2f6ky59IK01vpGFpUd4x_QX6zVs0xQnGFEWnSdgaeLM=232)

**Author’s Reply:** The manuscript has been uploaded along with other necessary documents following PLOS ONE’s manuscript submission guideline.

2. When completing the data availability statement of the submission form, you indicated that you will make your data available on acceptance. We strongly recommend all authors decide on a data sharing plan before acceptance, as the process can be lengthy and hold up publication timelines. Please note that, though access restrictions are acceptable now, your entire data will need to be made freely accessible if your manuscript is accepted for publication. This policy applies to all data except where public deposition would breach compliance with the protocol approved by your research ethics board. If you are unable to adhere to our open data policy, please kindly revise your statement to explain your reasoning and we will seek the editor's input on an exemption. Please be assured that, once you have provided your new statement, the assessment of your exemption will not hold up the peer review process.

**Author’s Reply:** The statement has been revised to, “All relevant data are within the manuscript and its Supporting Information files.”

3. We note that there is identifying data in the Supporting Information file < S1 File.xlsx>. Due to the inclusion of these potentially identifying data, we have removed this file from your file inventory. Prior to sharing human research participant data, authors should consult with an ethics committee to ensure data are shared in accordance with participant consent and all applicable local laws.

Data sharing should never compromise participant privacy. It is therefore not appropriate to publicly share personally identifiable data on human research participants. The following are examples of data that should not be shared:

-Name, initials, physical address

-Ages more specific than whole numbers

-Internet protocol (IP) address

-Specific dates (birth dates, death dates, examination dates, etc.)

-Contact information such as phone number or email address

-Location data

-ID numbers that seem specific (long numbers, include initials, titled “Hospital ID”) rather than random (small numbers in numerical order)

Data that are not directly identifying may also be inappropriate to share, as in combination they can become identifying. For example, data collected from a small group of participants, vulnerable populations, or private groups should not be shared if they involve indirect identifiers (such as sex, ethnicity, location, etc.) that may risk the identification of study participants.

Additional guidance on preparing raw data for publication can be found in our Data Policy (<https://journals.plos.org/plosone/s/data-availability#loc-human-research-participant-data-and-other-sensitive-data>) and in the following article: http://www.bmj.com/content/340/bmj.c181.long.

Please remove or anonymize all personal information (<specific identifying information in file to be removed>), ensure that the data shared are in accordance with participant consent, and re-upload a fully anonymized data set. Please note that spreadsheet columns with personal information must be removed and not hidden as all hidden columns will appear in the published file.

**Author’s Reply:** We thank the editor for this suggestion. We have reviewed the dataset and removed all personalized information.

4. We notice that your supplementary figure is uploaded with the file type 'Figure'. Please amend the file type to 'Supporting Information'. Please ensure that each Supporting Information file has a legend listed in the manuscript after the references list.

**Author’s Reply:** Thank you for the comment. We have amended the supplementary figure file type from “Figure” to “Supporting Information” in the submission system. We have also added legends for each Supporting Information file after the References list in the manuscript.

If the reviewer comments include a recommendation to cite specific previously published works, please review and evaluate these publications to determine whether they are relevant and should be cited. There is no requirement to cite these works unless the editor has indicated otherwise.

Please review your reference list to ensure that it is complete and correct. If you have cited papers that have been retracted, please include the rationale for doing so in the manuscript text, or remove these references and replace them with relevant current references. Any changes to the reference list should be mentioned in the rebuttal letter that accompanies your revised manuscript. If you need to cite a retracted article, indicate the article’s retracted status in the References list and also include a citation and full reference for the retraction notice.

**Author’s Reply:** Thank you for the comment. We have amended the file type to ‘Supporting Information’ (S2 File). We have carefully reviewed the reference list to ensure completeness and accuracy. No retracted articles were cited in the manuscript. All references have been checked for correctness, and DOIs have been verified. Any changes made to the reference list during revision are reflected in the updated manuscript.

**5. Review Comments to the Author**

Please use the space provided to explain your answers to the questions above. You may also include additional comments for the author, including concerns about dual publication, research ethics, or publication ethics. (Please upload your review as an attachment if it exceeds 20,000 characters)

**Reviewer #1**

The purpose of this study was to assess the prevalence of nomophobia among Bangladeshi medical and university students as well as look into the sociodemographic characteristics and smartphone-related behaviors that are linked to it. My observations are given below:

(1) The study title is okay.

**Author’s Reply**: We thank the reviewer for this comment.

(2) The abstract needs minor revision. It is thorough and up to date, with precise goals and suitable techniques. Enhance concision, provide justification for a longer data collection period, elucidate the sampling strategy, report effect sizes, temper causal language given the cross-sectional design, and contextualize the findings within Bangladesh.

**Author’s Reply:** We thank the reviewer for this comment. We have edited the abstract to improve concision and clarity. In particular, we clarified the longer data collection period by indicating that data was collected from September 2023 to July 2025 to capture diversity across academic cycles and to ensure wider geographic representation. We also clarified the sampling strategy by indicating that participants were recruited using non-probability snowball sampling through peer networks and institutional connections. Additionally, effect sizes were added as β coefficients with 95% confidence intervals and causal language was softened using association-based language appropriate for the cross-sectional study design. The conclusion of the abstract was also edited to explicitly state that causality cannot be inferred and to contextualize the findings within the socio-cultural and digital context of Bangladesh. The revised manuscript has been modified as follows. **Page 2, Lines 36-38, 43-45, 48-49; Page 3, Lines 51-54, 60-61, 63-64.**

(3) The introduction gives a general overview of nomophobia and references a lot of research, but it needs a lot of work. Prevalence statistics and definitions are occasionally out-of-date or geographically misaligned, and the justification for Bangladesh's focus seems dated and poorly thought out. Numerous sentences are repetitive, too detailed, or only loosely related. Citations should not be listed; instead, they should be condensed and critically synthesized. The theoretical framework lacks clarity, and the main research gaps are not stated clearly. The introduction would be strengthened by making concepts clearer, tightening the structure, and enhancing coherence.

**Author’s Reply**: Thank you for this valuable comment. We have revised the Introduction substantially to improve its clarity, coherence and critical synthesis. The definition of nomophobia was refined by framing it not only as fear or distress related to lack of mobile phone access but also within the broader context of behavioral addiction and problematic smartphone use. We updated and geographically aligned the background by adding recent global and Bangladesh-specific mobile phone subscription statistics. We also condensed the citation-heavy discussion by synthesizing previous evidence on demographic factors, gender, age, duration of phone use, anxiety, depression, and prevalence estimates, rather than listing studies individually. The theoretical framework was clarified. Finally, the research gap was clearly articulated by emphasizing the lack of Bangladesh-specific evidence, particularly regarding medical versus non-medical students and smartphone-related behavioral correlates within a unified analytical framework. The edits have been made in the revised manuscript as follows: **Page 3, Lines 69–70, 72-73; Page 4, Lines 79-80,84-86, 90-91,94-95; Page 5, Lines 98-99, 103-107**.

(4) Although the methodology is generally clear, there are a few issues that need to be addressed. It should be noted that selection bias may be introduced and generalizability may be limited by snowball sampling and online data collection. For a cross-sectional design, the two-year data collection window is excessively long and requires explanation. There are reported conflicting NMP-Q cut-off classifications that need to be resolved. Regression, ANOVA, and t-test assumptions are not specified. Clarification is needed regarding the operational definitions of independent variables and how to handle missing data. There is no power analysis or sample size calculation.

**Author’s Reply**:

Thank you for this valuable comment. We have revised the Methodology section to address all the concerns raised. First, we clarified the study design and sampling procedure, including the use of snowball sampling among undergraduate students from eight districts/divisions of Bangladesh. We also added explanation regarding the longer data collection period, stating the 22-month duration was due to the need to capture heterogeneity across academic cycles, institutional calendars and examination phases, as well as the snowball nature of sampling, resource constraints and the effort to recruit participants from all eight divisions of Bangladesh. We have added detailed sample size calculation using the single proportion formula with assumed prevalence, 95% confidence level, 5% margin of error, 15% non-response adjustment, final sample size, number approached, number included and number excluded due to incomplete responses. We have also clarified the eligibility criteria, pilot testing and reliability of the questionnaire. To resolve the conflicting classifications of NMP-Q cut-off scores, we clarified that our study adopted the widely accepted classification of 20 = absence, 21–59 = mild, 60–99 = moderate, and ≥100 = severe nomophobia, while acknowledging the existence of alternative classifications. We also explained the assessment of independent variables, including sociodemographic characteristics, device type, application/social media use, duration of daily phone use, phone use before sleep, and phone checking after waking. In the Statistical Analysis section, assumptions for t-test, ANOVA, and regression were specified and missing data were handled using complete-case analysis, as the proportion of missing data was small. Changes have been made in the revised manuscript as follows: **Page 5, Lines: 117-122; Page 6, Lines: 126-130, 138-139; Page 7-8, Lines: 148-151, 156-174.**

(5) With thorough descriptive statistics and appropriate use of bivariate and multivariate analyses, the results are thorough and presented in an understandable manner. Nevertheless, the sample size reporting is inconsistent (n=476 vs. n=436) that need to be clarified. There are apparent typographical errors in certain tables (e.g. frequencies of the academic year). It would improve interpretation if effect sizes were reported in addition to p-values. Think about eliminating unnecessary repetition in the text and tables and providing clear justification for the covariates used in regression models. Additionally, to further improve methodological transparency, assumptions testing and handling missing data should be reported.

**Author’s Reply**: We thank you for this valuable comment. We have made the following changes in the Results and Statistical Analysis sections to address these issues. First, we have corrected the inconsistent sample size reporting throughout the manuscript and the final analytic sample is now consistently reported as n = 436. The typographical error in Table 1 related to the academic year frequency/percentage has also been corrected. To enhance the interpretation beyond the p-values, we have clearly presented the adjusted effect estimates from the multiple linear regression model as β coefficients with 95% confidence intervals in Table 3 and described the magnitude and direction of these associations in the Results section. We have added the effect size in the Table 2 for better interpretation. We further minimized overlap between the text and tables by discussing only main findings in the text, with detailed distributions in the tables, and we explained the reasons for covariate selection in the regression model, which was mainly based on theoretical relevance and previous literature, and kept household income because it was found to be statistically significantly associated with nomophobia in bivariate analysis at p < 0.05. To enhance methodological transparency, we also specified assumptions testing for parametric tests and regression models and clarified that missing data were minimal and handled using complete-case analysis. The changes were made in the revised manuscript as follows: **Page 8, Lines 182; Page 12, Lines 209-211; Page 16, Line 228-235; Page 17, Lines 240-241, 244, 247-250; Page 18, Lines 254-255; Table 3**.

(6) The thorough and properly referenced discussion successfully places the findings in both regional and global literature. But instead of providing a more thorough theoretical interpretation, it is repetitive and unduly descriptive, frequently restating the findings. Age and gender comparisons are erratic and require more precise synthesis. The conclusion could be shorter and more focused on prevention or intervention implications rather than generalizations. A greater focus should be placed on the limitations of sampling and the use of self-report.

**Author’s Reply**: Thanks for the valuable comment. We have revised the Discussion and Conclusion sections to reduce repetition, strengthen theoretical interpretation, improve synthesis of age and gender-related findings, and focus the conclusion more clearly on prevention and intervention implications. Firstly, we added a theoretical interpretation of the findings by linking higher nomophobia scores with behavioral dependence, cognitive salience, habitual smartphone checking, and anxiety related to disconnection. This was done to move the discussion beyond descriptive comparison with previous studies. We also added citations to support this theoretical interpretation. Secondly, we revised the discussion of age and gender for a more balanced synthesis. We made it clear that age-related findings are inconsistent between studies and should be interpreted with caution. For gender, we provided a more detailed explanation for the possible increased vulnerability of female students to nomophobia, including the greater use of smartphones for social networking, emotional communication, reassurance, maintenance of interpersonal relationships, and anxiety-related vulnerability. We also acknowledged that some studies report higher nomophobia among males, and that gender-related findings may vary depending on smartphone-use motives, sociocultural context, and behavioral patterns. Third, we revised the discussion to emphasize that nomophobia is a complex interaction of behavioral habits, psychological dependency, and environmental exposure and not just device availability. We also improved the limitations section by emphasizing the limitations of cross-sectional design, self-reported online data, recall and social desirability bias, snowball sampling, selection bias, limited generalizability, and residual confounding by unmeasured psychological factors. Finally, the conclusion was revised to add more emphasis on preventive and behavioral interventions, such as digital literacy, behavioral awareness, structured healthy smartphone use guidelines, and institutional mental well-being policies. The revised manuscript has been modified as follows: **Page 18-19, Lines 265-269; Page 19-20, Lines 282-285, 287-311; Page 21, Lines 324-334, 340-341; Page 22, Lines 343-344; Page 22, Lines 355-357**; **Page 22-23, Lines 367-372.**

(7) The references are okay. Please correct it by following the journal’s guidelines. The tables and figures should be prepared following standard guidelines.

**Author’s Reply:** Thank you for your precious comment. We have followed the journal’s guidelines and tables and figures are prepared by following standard guidelines.

**Reviewer #2:**

Based on the title, there are differences between medical students and other academic specializations, but there are no clear indications of these differences in the paper's results or discussion; the authors failed to use this particularity statistically.

**Author’s Reply:** Thank you for this valuable comment. We clarified that academic specialization was categorized as medical and non-medical students and included as an independent variable. The distribution of medical and non-medical students is presented in Table 1, and their bivariate comparison with NMP-Q scores is presented in Table 2. The results showed no statistically significant difference in mean NMP-Q scores between medical and non-medical students. Therefore, we revised the Discussion to state that academic specialization was considered as a potential determinant but did not show a significant independent association after adjustment, suggesting that nomophobia may be more strongly related to smartphone-use behaviors than academic discipline alone. We also included this as a limitation because broad categorization of medical versus non-medical students may not capture nuanced differences across specific academic disciplines. Academic specialization was specifically mentioned in the revised manuscript on **Page 2, Lines 35–36; Page 5, Lines 106–107.**

It is appropriate to use this type of regression analysis with this data, but the authors did not specify whether the 'normality of residuals' or 'homoscedasticity' were tested, both of which are essential for the validity of linear regression results. The absence of the coefficient of determination (R²) means the overall 'power of the model' was not provided; its absence leaves us uncertain whether the model explains only 10% or 70% of the problem. Additionally, the paper included 'age' in the model despite its very weak correlation (r=0.12).

**Author’s Reply:** Thank you for the comments. We conducted the Breusch-Pagan test (p-value=0.99) to confirm homoscedasticity. Normality of residuals was verified via Shapiro-Wilk test (p-value=0.27). The co-efficient of determination of this model was 0.0725 indicating that the model explains roughly 7% of the variance in total NMP score. The primary goal of this analysis was to identify statistically significant associations rather than to achieve high predictive power. Given the complexity of Nomophobia, it is expected that other unobserved factors contribute to the remaining variance. However, the significance of our predictors remains robust, and the model diagnostics indicate that the linear regression assumptions are satisfied. Additionally, we would like to clarify that age was not included in the adjusted model presented in Table 3. In the paragraph preceding Table 3, we discussed the weak correlation between age and nomophobia scores; however, age was not incorporated into the model. We recognize that mentioning age in that section may have created the impression that it was included in the analysis. To address this and clearly specify the variables included in the model for Table 3, we have added a clarifying sentence on **Page 17, Lines 248–250.**

Some of these variables could have been considered mediating variables (e.g., social media applications, daily usage hours), and mediation analysis could have been used to interpret the results more accurately.

**Author’s Reply:** Thank you for the valuable comment. We agree that variables such as social media/communication application use and daily smartphone-use duration could be mediating variables. However, we did not perform mediation analysis because the cross-sectional nature of the study limits temporal inference and does not allow clear establishment of the exposure–mediator–outcome sequence required for meaningful mediation analysis. We have now added this as a limitation in the manuscript. The revised manuscript has been modified as follows: **Page 22, Lines 354-358;**

Writing all demographic and descriptive data in a long textual format makes it merely a textual account of the numbers. Furthermore, the tables need formatting as they contain too much information.

**Author’s Reply:** Thank you for your helpful comment. We have edited the Results section to reduce the duplication of data in the text and tables. We no longer include all demographic and descriptive values in the text but have kept the detailed distributions in Table 1 with a summary of the key descriptive findings in the text. We also added a sentence to the Results section, to be clearer, that the key findings are summarized in the text and the detailed distributions are shown in the tables to improve readability and avoid repetition. In addition, table formatting was reviewed and typographical errors in Table 1 (eg, academic year percentage/frequency error) were corrected. The revised manuscript has been modified as follows: **Page 9, Table 1; Page 12, Lines 209-211; Page 16, Lines 228-235; Page 17, Lines 240-241, 244, 248-250; Page 17, Table 3; Page 18, Lines 254-255.**

In lines (168-179), the researcher used complex and repetitive technical language. An introductory paragraph could have been written saying: 'After adjusting for demographic variables (gender, income, etc.), the results showed the following...

**Author’s Reply:** We thank this helpful suggestion. We have made some modifications in the interpretation paragraph before Table 3 to improve clarity, reduce repetition and facilitate understanding. We have added an introductory sentence before presenting the adjusted regression results, clarifying that estimates were obtained from a multiple linear regression model with simultaneous adjustment for gender, income, social networking/communication application use, average daily phone use and checking the phone immediately upon waking and simplified the interpretation of each adjusted association and reduced the use of repeated technical wording throughout the paragraph. The revised manuscript has been modified as follows: **Page 16, Lines 228-235; Page 17, Lines 240-241; Page 17, Lines 247-250.**

The discussion section exaggerates the statistical comparison between the study's results and other papers without providing a clear benefit for discussing the findings. For instance, the authors did not provide any explanation in the discussion for why females are more prone to nomophobia.

**Author’s Reply:**

Thank you for this valuable comment. We revised the Discussion section to reduce overemphasis on simple statistical comparison with previous studies and to provide a clearer theoretical and behavioral interpretation of the findings. Specifically, we added an explanatory framework linking nomophobia with behavioral dependence, cognitive salience, habitual checking behavior, and anxiety related to disconnection. This was done to interpret the findings beyond descriptive comparison with regional and global studies. We also substantially revised the age and gender discussion. Instead of only stating that females were more prone to nomophobia, we added possible explanations from the literature. We explained that female students may be more vulnerable because they may use smartphones more frequently for social networking, emotional communication, reassurance, and maintaining interpersonal relationships. We further added that dependence on smartphones for relational and emotional communication may increase anxiety when disconnected from the device. We also linked nomophobia with fear of missing out, fear of losing communication access, psychological distress, and the higher burden of anxiety disorders among females. To avoid overgeneralization, we acknowledged that gender-related findings are mixed across studies and should be interpreted within the sociocultural and behavioral context of the study population. The revisions are made in the revised manuscript as follows: **Page 18-19, Lines 265-269; Page 19, Lines 282-285; Page 19-20, Lines 287-311; Page 21, Lines 324-334, 340-341; Page 22, Line 343-344, 354-358, 359-361, 363-365.**

In a nutshell, nomophobia is a pressing issue in today’s world. In this modern era, avoiding smartphones and devices is nearly impossible as we are heavily dependent on them in our day-to-day activities. Therefore, it is crucial to raise awareness of the negative impacts of nomophobia and conduct extensive research on it.

**Author’s Reply:** Thank you for your valuable comment. The conclusion section has been rephrased to be concise and targeted, and less broad. Instead of ending with a generic statement that nomophobia is an issue of modern age, we have provided more specific implications for prevention and intervention. Specifically, the revised conclusion underscores the importance of targeted preventative and behavioral interventions such as digital literacy programs, behavioral awareness activities, structured guidelines for healthy smartphone usage, institutional policies that promote a balanced use of technology, and future longitudinal studies to strengthen causal inference. The revisions are made in the revised manuscript as follows: **Page 23, Lines 371-376.**
