# Supplementary material for: Prevalence and associated factors of nomophobia among the medical and university students of Bangladesh
Source: PLoS One. 2026 Aug 31;21(8):e0341524. doi: 10.1371/journal.pone.0341524 (PMC13528988; doi:10.1371/journal.pone.0341524)
Supplement: Supplementary file 2 [file pone.0341524.s004.docx]

**Response to Reviewer’s Comments**

**Journal Requirements:**

**Editorial Comments:**

1. Please check the punctuation mark and any grammatical error.

'Phone use upon waking, and extensive engagement with social communication and media applications are associated with excessive smartphone use and the development of nomophobia; however, causality cannot be inferred due to the cross-sectional study design.”

 The semicolon before “however” is missing and should be inserted.

**Author’s Reply:** We thank the academic editor for pointing this out. We have revised the sentence by inserting a semicolon before “however” and a comma after “however.” We also removed the unnecessary comma after “waking” and added “the” before “development” to improve grammatical accuracy and readability. This correction has been made in the conclusion section of the abstract. The revised manuscript has been modified as follows: **Page 3, Lines 59, 60; Page 7, Lines 150-151 161, 163-164; Page 13, Lines 243.**

2. Please avoid excessive multiple citations supporting a single statement in the introduction. In the sentence:

             Relevant text (“Although no notable gender differences were found in smartphone use, nomophobia scores between men and women showed remarkable deviation [14,17,18,20–24,26,30–32]”.)

 The large number of references should be reduced and only the most relevant and representative studies should be cited to improve readability and avoid citation clutter.

**Author’s Reply:** Thank you for this helpful suggestion. We agree that the previous sentence contained excessive citations supporting a single statement, which reduced readability. To address this, we have revised the sentence by breaking it into multiple sentences and retaining only the most relevant and representative references for each specific claim. The revised manuscript has been modified as follows: **Page 4, Lines 90-94.**

3. Please clarify the exclusion criteria. The manuscript states that students who did not own a mobile device were excluded. However, it is unclear whether this refers to students without any mobile phone or specifically those without smartphones.

**Author’s Reply:** Thank you for pointing out this ambiguity. We have clarified the exclusion criteria in the Methods section. The exclusion criterion referred to students who did not own any type of personal mobile phone, including button phones, multimedia/feature phones, or smartphones as well as any type of digital devices. The revised manuscript has been modified as follows. **Page 6, Lines 132-136.**

4.  The authors have not clearly stated whether the Nomophobia Questionnaire (NMP-Q) has been previously validated in the Bangladeshi population. The manuscript only mentions a pilot test. This issue should be addressed in the limitations section. The authors should also discuss the absence of formal psychometric validation (e.g., construct validity assessment) or provide additional validation analyses if available. [Relevant text:“Prior to the main study, the questionnaire underwent a pilot test among 60 participants to assess reliability using Cronbach’s alpha (α = 0.82).]

**Author’s Reply:** Thank you for this important comment. We have revised the Methods section to clarify the use of the NMP-Q and to distinguish reliability assessment from formal psychometric validation. The questionnaire was pilot-tested in the present study and showed good internal consistency reliability, with a Cronbach’s alpha of 0.82. However, we acknowledge that no formal construct validity assessment, such as exploratory or confirmatory factor analysis, was conducted in our sample. We have therefore added this issue to the Limitations section and interpreted the findings accordingly. The revised manuscript has been modified as follows: **Page 6, Lines 137-142, Page 23, Lines 391-399.**

5. In line 207, please replace “higher nomophobia” with “higher nomophobia score” for clarity and scientific accuracy.

**Author’s Reply:** Thank you for this suggestion. We have replaced “higher nomophobia” with “higher nomophobia scores” to improve clarity and scientific accuracy. The revised manuscript has been modified as follows: **Page 13, Line 219.**

6.Several reported effect sizes (e.g., η² = 0.015) are marked. Please verify whether these have been correctly interpreted and whether they are statistically and practically meaningful. Ensure consistency in reporting and interpretation of effect sizes throughout the manuscript.

**Author’s Reply:** Thank you for this important comment. We have rechecked the reported effect sizes and revised the interpretation throughout the manuscript. We clarified that statistically significant findings with small η² values, such as η² = 0.015, indicate small practical effects despite statistical significance. We also added a table footnote describing the interpretation thresholds for Cohen’s d and η² to ensure consistency in reporting. Effect sizes are now interpreted cautiously and are not overstated as practically meaningful when their magnitude is small. The revised manuscript has been modified as follows. During rechecking, we identified an incorrectly reported η² value for ‘Via Mobile Data.’ Since η² values must range between 0 and 1, this value was recalculated and corrected in Table 2. The revised manuscript has been modified as follows: **Page 13, Lines 225-233; Table 2; Page 17, Lines 239-241.**

7. The statistical analysis section requires revision for consistency. The authors state that both multiple linear regression and logistic regression were used; however, the outcome variable (NMP-Q score) is continuous, and only multiple linear regression is presented in the results. No logistic regression analysis or corresponding results are provided in the manuscript. Therefore, the mention of logistic regression is misleading and should be removed or revised to accurately reflect the analysis performed. The authors should ensure that the statistical methods section is fully consistent with the reported analyses. Additionally, the description of regression assumption testing is overly detailed and should be streamlined to remove redundant wording.

**Author’s Reply:** Thank you for this important observation. We agree that the previous Statistical analysis section was inconsistent with the analyses reported in the Results section. Since the outcome variable was the continuous NMP-Q total score and only multiple linear regression was presented, we have removed the references to logistic regression. The Statistical analysis section has been revised to accurately describe the analyses performed, including t-tests, ANOVA, Pearson correlation, and multiple linear regression. We have also streamlined the description of regression assumption testing to avoid redundant wording. The revised manuscript has been modified as follows: **Page 7, Lines 161, 163-165; Page 8, Lines 171-185.**

**Comments by Trisha Mae Tañedo Perez**

1. Please include a legend for figure 1.

**Author’s Reply:** We thank the editor for this important observation. We have added a legend for figure 1. The revised manuscript has been modified as follows in the tracked change version: **Page 13, Lines 239-245.**

2. Please upload a copy of Figure 1 which you refer to in your text on page 12. Or if the figure is no longer to be included as part of the submission, please remove all reference to it within the text.

**Author’s Reply:** Thank you for this important suggestion. We have uploaded a copy of Figure 1 as follows: **Figure “Fig 1.tiff” and Supporting Information “S2 File_5.21.26.tiff”.**

**6. Review Comments to the Author**

**Reviewer #1**

I thoroughly examined the updated manuscript and discovered that the authors had successfully incorporated the recommendations from the earlier review. It does not require further changes.

**Author’s Reply:** We sincerely thank the reviewer for carefully re-evaluating the revised manuscript and for acknowledging that the previous recommendations have been satisfactorily addressed. We are grateful for the reviewer’s constructive comments, which helped improve the clarity, consistency, and overall quality of the manuscript.

**Reviewer #2**

 I would like to thank the authors for their meticulous efforts in thoroughly addressing all my concerns in this revision. The statistical rigor has been significantly improved by properly testing the regression assumptions and including the R² value. Furthermore, the distinction between medical and non-medical specializations is now clear, and the data presentation has been streamlined beautifully. The discussion section provides meaningful, well-reasoned socio-behavioral explanations for the findings. The manuscript now fully meets the high standards of PLOS ONE. I recommend it for final acceptance.

**Author’s Reply:** We sincerely thank the reviewer for the careful re-evaluation of our revised manuscript and for the generous and encouraging feedback. We are grateful that the reviewer found the revisions satisfactory, particularly regarding the improved statistical reporting, clarification of academic specialization, streamlined data presentation, and strengthened discussion. The reviewer’s thoughtful comments throughout the review process were invaluable in improving the clarity, rigor, and overall quality of the manuscript. We deeply appreciate the recommendation for final acceptance.
