# Supplementary material for: Prevalence and associated factors of nomophobia among the medical and university students of Bangladesh
Source: PLoS One. 2026 Aug 31;21(8):e0341524. doi: 10.1371/journal.pone.0341524 (PMC13528988; doi:10.1371/journal.pone.0341524)
Supplement: Peer review history

# Peer review: Decision Letter 0

**DOI:** 10.1371/journal.pone.0341524.r001

**Authors:** Syeda Humaida Hasan

25 Feb 2026

Dear Dr. Chowdhury,

Thank you for submitting your manuscript to PLOS ONE. After careful consideration, we feel that it has merit but does not fully meet PLOS ONE’s publication criteria as it currently stands. Therefore, we invite you to submit a revised version of the manuscript that addresses the points raised during the review process.

Please submit your revised manuscript by Apr 11 2026 11:59PM. If you will need more time than this to complete your revisions, please reply to this message or contact the journal office at plosone@plos.org. When you're ready to submit your revision, log on to https://www.editorialmanager.com/pone/ and select the 'Submissions Needing Revision' folder to locate your manuscript file.

- A letter that responds to each point raised by the academic editor and reviewer(s). You should upload this letter as a separate file labeled 'Response to Reviewers'.
- A marked-up copy of your manuscript that highlights changes made to the original version. You should upload this as a separate file labeled 'Revised Manuscript with Track Changes'.
- An unmarked version of your revised paper without tracked changes. You should upload this as a separate file labeled 'Manuscript'.

If applicable, we recommend that you deposit your laboratory protocols in protocols.io to enhance the reproducibility of your results. Protocols.io assigns your protocol its own identifier (DOI) so that it can be cited independently in the future. For instructions see: https://journals.plos.org/plosone/s/submission-guidelines#loc-laboratory-protocols. Additionally, PLOS ONE offers an option for publishing peer-reviewed Lab Protocol articles, which describe protocols hosted on protocols.io. Read more information on sharing protocols at https://plos.org/protocols?utm_medium=editorial-email&utm_source=authorletters&utm_campaign=protocols.

We look forward to receiving your revised manuscript.

Kind regards,

Syeda Humaida Hasan, Diploma in Child Health, FCPS (Pediatrics)

Academic Editor

PLOS One

Journal requirements:

When submitting your revision, we need you to address these additional requirements.

1. Please ensure that your manuscript meets PLOS ONE's style requirements, including those for file naming. The PLOS ONE style templates can be found at

https://journals.plos.org/plosone/s/file?id=wjVg/PLOSOne_formatting_sample_main_body.pdf and

https://journals.plos.org/plosone/s/file?id=ba62/PLOSOne_formatting_sample_title_authors_affiliations.pdf

2. When completing the data availability statement of the submission form, you indicated that you will make your data available on acceptance. We strongly recommend all authors decide on a data sharing plan before acceptance, as the process can be lengthy and hold up publication timelines. Please note that, though access restrictions are acceptable now, your entire data will need to be made freely accessible if your manuscript is accepted for publication. This policy applies to all data except where public deposition would breach compliance with the protocol approved by your research ethics board. If you are unable to adhere to our open data policy, please kindly revise your statement to explain your reasoning and we will seek the editor's input on an exemption. Please be assured that, once you have provided your new statement, the assessment of your exemption will not hold up the peer review process.

3. We note that there is identifying data in the Supporting Information file < S1 File.xlsx>. Due to the inclusion of these potentially identifying data, we have removed this file from your file inventory. Prior to sharing human research participant data, authors should consult with an ethics committee to ensure data are shared in accordance with participant consent and all applicable local laws.

Data sharing should never compromise participant privacy. It is therefore not appropriate to publicly share personally identifiable data on human research participants. The following are examples of data that should not be shared:

-Name, initials, physical address

-Ages more specific than whole numbers

-Internet protocol (IP) address

-Specific dates (birth dates, death dates, examination dates, etc.)

-Contact information such as phone number or email address

-Location data

-ID numbers that seem specific (long numbers, include initials, titled “Hospital ID”) rather than random (small numbers in numerical order)

Data that are not directly identifying may also be inappropriate to share, as in combination they can become identifying. For example, data collected from a small group of participants, vulnerable populations, or private groups should not be shared if they involve indirect identifiers (such as sex, ethnicity, location, etc.) that may risk the identification of study participants.

Additional guidance on preparing raw data for publication can be found in our Data Policy (https://journals.plos.org/plosone/s/data-availability#loc-human-research-participant-data-and-other-sensitive-data) and in the following article: http://www.bmj.com/content/340/bmj.c181.long.

Please remove or anonymize all personal information (<specific identifying information in file to be removed>), ensure that the data shared are in accordance with participant consent, and re-upload a fully anonymized data set. Please note that spreadsheet columns with personal information must be removed and not hidden as all hidden columns will appear in the published file.

4. We notice that your supplementary figure is uploaded with the file type 'Figure'. Please amend the file type to 'Supporting Information'. Please ensure that each Supporting Information file has a legend listed in the manuscript after the references list.

If the reviewer comments include a recommendation to cite specific previously published works, please review and evaluate these publications to determine whether they are relevant and should be cited. There is no requirement to cite these works unless the editor has indicated otherwise.

Please review your reference list to ensure that it is complete and correct. If you have cited papers that have been retracted, please include the rationale for doing so in the manuscript text, or remove these references and replace them with relevant current references. Any changes to the reference list should be mentioned in the rebuttal letter that accompanies your revised manuscript. If you need to cite a retracted article, indicate the article’s retracted status in the References list and also include a citation and full reference for the retraction notice.

[Note: HTML markup is below. Please do not edit.]

Reviewers' comments:

Reviewer's Responses to Questions

**Comments to the Author**

1. Is the manuscript technically sound, and do the data support the conclusions?

Reviewer #1: Yes

Reviewer #2: Yes

**********

2. Has the statistical analysis been performed appropriately and rigorously?

Reviewer #1: Yes

Reviewer #2: Yes

**********

3. Have the authors made all data underlying the findings in their manuscript fully available?

The PLOS Data policy

Reviewer #1: Yes

Reviewer #2: Yes

**********

4. Is the manuscript presented in an intelligible fashion and written in standard English?

Reviewer #1: Yes

Reviewer #2: Yes

**********

Reviewer #1: The purpose of this study was to assess the prevalence of nomophobia among Bangladeshi medical and university students as well as look into the sociodemographic characteristics and smartphone-related behaviours that are linked to it. My observations are given below:

(1) The study title is okay.

(2) The abstract needs minor revision. It is thorough and up to date, with precise goals and suitable techniques. Enhance concision, provide justification for a longer data collection period, elucidate the sampling strategy, report effect sizes, temper causal language given the cross-sectional design, and contextualize the findings within Bangladesh.

(3) The introduction gives a general overview of nomophobia and references a lot of research, but it needs a lot of work. Prevalence statistics and definitions are occasionally out-of-date or geographically misaligned, and the justification for Bangladesh's focus seems dated and poorly thought out. Numerous sentences are repetitive, too detailed, or only loosely related. Citations should not be listed; instead, they should be condensed and critically synthesized. The theoretical framework lacks clarity, and the main research gaps are not stated clearly. The introduction would be strengthened by making concepts clearer, tightening the structure, and enhancing coherence.

(4) Although the methodology is generally clear, there are a few issues that need to be addressed. It should be noted that selection bias may be introduced and generalizability may be limited by snowball sampling and online data collection. For a cross-sectional design, the two-year data collection window is excessively long and requires explanation. There are reported conflicting NMP-Q cut-off classifications that need to be resolved. Regression, ANOVA, and t-test assumptions are not specified. Clarification is needed regarding the operational definitions of independent variables and how to handle missing data. There is no power analysis or sample size calculation.

(5) With thorough descriptive statistics and appropriate use of bivariate and multivariate analyses, the results are thorough and presented in an understandable manner. Nevertheless, the sample size reporting is inconsistent (n=476 vs. n=436) that need to be clarified. There are apparent typographical errors in certain tables (e.g. frequencies of the academic year). It would improve interpretation if effect sizes were reported in addition to p-values. Think about eliminating unnecessary repetition in the text and tables and providing clear justification for the covariates used in regression models. Additionally, to further improve methodological transparency, assumptions testing and handling missing data should be reported.

(6) The thorough and properly referenced discussion successfully places the findings in both regional and global literature. But instead of providing a more thorough theoretical interpretation, it is repetitive and unduly descriptive, frequently restating the findings. Age and gender comparisons are erratic and require more precise synthesis. The conclusion could be shorter and more focused on prevention or intervention implications rather than generalizations. A greater focus should be placed on the limitations of sampling and the use of self-report.

(7) The references are okay. Please correct it by following the journal’s guidelines. The tables and figures should be prepared following standard guidelines.

Reviewer #2: Based on the title, there are differences between medical students and other academic specializations, but there are no clear indications of these differences in the paper's results or discussion; the authors failed to use this particularity statistically.

It is appropriate to use this type of regression analysis with this data, but the authors did not specify whether the 'normality of residuals' or 'homoscedasticity' were tested, both of which are essential for the validity of linear regression results. The absence of the coefficient of determination (R²) means the overall 'power of the model' was not provided; its absence leaves us uncertain whether the model explains only 10% or 70% of the problem. Additionally, the paper included 'age' in the model despite its very weak correlation (r=0.12).

Some of these variables could have been considered mediating variables (e.g., social media applications, daily usage hours), and mediation analysis could have been used to interpret the results more accurately.

Writing all demographic and descriptive data in a long textual format makes it merely a textual account of the numbers. Furthermore, the tables need formatting as they contain too much information.

In lines (168-179), the researcher used complex and repetitive technical language. An introductory paragraph could have been written saying: 'After adjusting for demographic variables (gender, income, etc.), the results showed the following...

The discussion section exaggerates the statistical comparison between the study's results and other papers without providing a clear benefit for discussing the findings. For instance, the authors did not provide any explanation in the discussion for why females are more prone to nomophobia.

In a nutshell, nomophobia is a pressing issue in today’s world. In this modern era, avoiding smartphones and devices is nearly impossible as we are heavily dependent on them in our day-to-day activities. Therefore, it is crucial to raise awareness of the negative impacts of nomophobia and conduct extensive research on it.

**********

what does this mean?). If published, this will include your full peer review and any attached files.

If you choose “no”, your identity will remain anonymous but your review may still be made public.

**Do you want your identity to be public for this peer review?**  For information about this choice, including consent withdrawal, please see our Privacy Policy

Reviewer #1: **Yes:**  Gyanesh Kumar Tiwari

Reviewer #2: **Yes:**  Mahmud Hamakareem Mahmud

**********

[NOTE: If reviewer comments were submitted as an attachment file, they will be attached to this email and accessible via the submission site. Please log into your account, locate the manuscript record, and check for the action link "View Attachments". If this link does not appear, there are no attachment files.]

To ensure your figures meet our technical requirements, please review our figure guidelines: https://journals.plos.org/plosone/s/figures

You may also use PLOS’s free figure tool, NAAS, to help you prepare publication quality figures: https://journals.plos.org/plosone/s/figures#loc-tools-for-figure-preparation.

NAAS will assess whether your figures meet our technical requirements by comparing each figure against our figure specifications.

# Author comment: Author response to Decision Letter 1

**DOI:** 10.1371/journal.pone.0341524.r002

22 May 2026

Response to Reviewer’s Comments

Journal Requirements:

1. Please ensure that your manuscript meets PLOS ONE's style requirements, including those for file naming. The PLOS ONE style templates can be found at

https://journals.plos.org/plosone/s/file?id=wjVg/PLOSOne_formatting_sample_main_body.pdf and

https://journals.plos.org/plosone/s/file?id=ba62/PLOSOne_formatting_sample_title_authors_affiliations.pdf

Author’s Reply: The manuscript has been uploaded along with other necessary documents following PLOS ONE’s manuscript submission guideline.

2. When completing the data availability statement of the submission form, you indicated that you will make your data available on acceptance. We strongly recommend all authors decide on a data sharing plan before acceptance, as the process can be lengthy and hold up publication timelines. Please note that, though access restrictions are acceptable now, your entire data will need to be made freely accessible if your manuscript is accepted for publication. This policy applies to all data except where public deposition would breach compliance with the protocol approved by your research ethics board. If you are unable to adhere to our open data policy, please kindly revise your statement to explain your reasoning and we will seek the editor's input on an exemption. Please be assured that, once you have provided your new statement, the assessment of your exemption will not hold up the peer review process.

Author’s Reply: The statement has been revised to, “All relevant data are within the manuscript and its Supporting Information files.”

3. We note that there is identifying data in the Supporting Information file < S1 File.xlsx>. Due to the inclusion of these potentially identifying data, we have removed this file from your file inventory. Prior to sharing human research participant data, authors should consult with an ethics committee to ensure data are shared in accordance with participant consent and all applicable local laws.

Data sharing should never compromise participant privacy. It is therefore not appropriate to publicly share personally identifiable data on human research participants. The following are examples of data that should not be shared:

-Name, initials, physical address

-Ages more specific than whole numbers

-Internet protocol (IP) address

-Specific dates (birth dates, death dates, examination dates, etc.)

-Contact information such as phone number or email address

-Location data

-ID numbers that seem specific (long numbers, include initials, titled “Hospital ID”) rather than random (small numbers in numerical order)

Data that are not directly identifying may also be inappropriate to share, as in combination they can become identifying. For example, data collected from a small group of participants, vulnerable populations, or private groups should not be shared if they involve indirect identifiers (such as sex, ethnicity, location, etc.) that may risk the identification of study participants.

Additional guidance on preparing raw data for publication can be found in our Data Policy (https://journals.plos.org/plosone/s/data-availability#loc-human-research-participant-data-and-other-sensitive-data) and in the following article: http://www.bmj.com/content/340/bmj.c181.long.

Please remove or anonymize all personal information (<specific identifying information in file to be removed>), ensure that the data shared are in accordance with participant consent, and re-upload a fully anonymized data set. Please note that spreadsheet columns with personal information must be removed and not hidden as all hidden columns will appear in the published file.

Author’s Reply: We thank the editor for this suggestion. We have reviewed the dataset and removed all personalized information.

4. We notice that your supplementary figure is uploaded with the file type 'Figure'. Please amend the file type to 'Supporting Information'. Please ensure that each Supporting Information file has a legend listed in the manuscript after the references list.

Author’s Reply: Thank you for the comment. We have amended the supplementary figure file type from “Figure” to “Supporting Information” in the submission system. We have also added legends for each Supporting Information file after the References list in the manuscript.

If the reviewer comments include a recommendation to cite specific previously published works, please review and evaluate these publications to determine whether they are relevant and should be cited. There is no requirement to cite these works unless the editor has indicated otherwise.

Please review your reference list to ensure that it is complete and correct. If you have cited papers that have been retracted, please include the rationale for doing so in the manuscript text, or remove these references and replace them with relevant current references. Any changes to the reference list should be mentioned in the rebuttal letter that accompanies your revised manuscript. If you need to cite a retracted article, indicate the article’s retracted status in the References list and also include a citation and full reference for the retraction notice.

Author’s Reply: Thank you for the comment. We have amended the file type to ‘Supporting Information’ (S2 File). We have carefully reviewed the reference list to ensure completeness and accuracy. No retracted articles were cited in the manuscript. All references have been checked for correctness, and DOIs have been verified. Any changes made to the reference list during revision are reflected in the updated manuscript.

5. Review Comments to the Author

Please use the space provided to explain your answers to the questions above. You may also include additional comments for the author, including concerns about dual publication, research ethics, or publication ethics. (Please upload your review as an attachment if it exceeds 20,000 characters)

Reviewer #1

The purpose of this study was to assess the prevalence of nomophobia among Bangladeshi medical and university students as well as look into the sociodemographic characteristics and smartphone-related behaviors that are linked to it. My observations are given below:

(1) The study title is okay.

Author’s Reply: We thank the reviewer for this comment.

(2) The abstract needs minor revision. It is thorough and up to date, with precise goals and suitable techniques. Enhance concision, provide justification for a longer data collection period, elucidate the sampling strategy, report effect sizes, temper causal language given the cross-sectional design, and contextualize the findings within Bangladesh.

Author’s Reply: We thank the reviewer for this comment. We have edited the abstract to improve concision and clarity. In particular, we clarified the longer data collection period by indicating that data was collected from September 2023 to July 2025 to capture diversity across academic cycles and to ensure wider geographic representation. We also clarified the sampling strategy by indicating that participants were recruited using non-probability snowball sampling through peer networks and institutional connections. Additionally, effect sizes were added as β coefficients with 95% confidence intervals and causal language was softened using association-based language appropriate for the cross-sectional study design. The conclusion of the abstract was also edited to explicitly state that causality cannot be inferred and to contextualize the findings within the socio-cultural and digital context of Bangladesh. The revised manuscript has been modified as follows. Page 2, Lines 36-38, 43-45, 48-49; Page 3, Lines 51-54, 60-61, 63-64.

(3) The introduction gives a general overview of nomophobia and references a lot of research, but it needs a lot of work. Prevalence statistics and definitions are occasionally out-of-date or geographically misaligned, and the justification for Bangladesh's focus seems dated and poorly thought out. Numerous sentences are repetitive, too detailed, or only loosely related. Citations should not be listed; instead, they should be condensed and critically synthesized. The theoretical framework lacks clarity, and the main research gaps are not stated clearly. The introduction would be strengthened by making concepts clearer, tightening the structure, and enhancing coherence.

Author’s Reply: Thank you for this valuable comment. We have revised the Introduction substantially to improve its clarity, coherence and critical synthesis. The definition of nomophobia was refined by framing it not only as fear or distress related to lack of mobile phone access but also within the broader context of behavioral addiction and problematic smartphone use. We updated and geographically aligned the background by adding recent global and Bangladesh-specific mobile phone subscription statistics. We also condensed the citation-heavy discussion by synthesizing previous evidence on demographic factors, gender, age, duration of phone use, anxiety, depression, and prevalence estimates, rather than listing studies individually. The theoretical framework was clarified. Finally, the research gap was clearly articulated by emphasizing the lack of Bangladesh-specific evidence, particularly regarding medical versus non-medical students and smartphone-related behavioral correlates within a unified analytical framework. The edits have been made in the revised manuscript as follows: Page 3, Lines 69–70, 72-73; Page 4, Lines 79-80,84-86, 90-91,94-95; Page 5, Lines 98-99, 103-107.

(4) Although the methodology is generally clear, there are a few issues that need to be addressed. It should be noted that selection bias may be introduced and generalizability may be limited by snowball sampling and online data collection. For a cross-sectional design, the two-year data collection window is excessively long and requires explanation. There are reported conflicting NMP-Q cut-off classifications that need to be resolved. Regression, ANOVA, and t-test assumptions are not specified. Clarification is needed regarding the operational definitions of independent variables and how to handle missing data. There is no power analysis or sample size calculation.

Author’s Reply:

Thank you for this valuable comment. We have revised the Methodology section to address all the concerns raised. First, we clarified the study design and sampling procedure, including the use of snowball sampling among undergraduate students from eight districts/divisions of Bangladesh. We also added explanation regarding the longer data collection period, stating the 22-month duration was due to the need to capture heterogeneity across academic cycles, institutional calendars and examination phases, as well as the snowball nature of sampling, resource constraints and the effort to recruit participants from all eight divisions of Bangladesh. We have added detailed sample size calculation using the single proportion formula with assumed prevalence, 95% confidence level, 5% margin of error, 15% non-response adjustment, final sample size, number approached, number included and number excluded due to incomplete responses. We have also clarified the eligibility criteria, pilot testing and reliability of the questionnaire. To resolve the conflicting classifications of NMP-Q cut-off scores, we clarified that our study adopted the widely accepted classification of 20 = absence, 21–59 = mild, 60–99 = moderate, and ≥100 = severe nomophobia, while acknowledging the existence of alternative classifications. We also explained the assessment of independent variables, including sociodemographic characteristics, device type, application/social media use, duration of daily phone use, phone use before sleep, and phone checking after waking. In the Statistical Analysis section, assumptions for t-test, ANOVA, and regression were specified and missing data were handled using complete-case analysis, as the proportion of missing data was small. Changes have been made in the revised manuscript as follows: Page 5, Lines: 117-122; Page 6, Lines: 126-130, 138-139; Page 7-8, Lines: 148-151, 156-174.

(5) With thorough descriptive statistics and appropriate use of bivariate and multivariate analyses, the results are thorough and presented in an understandable manner. Nevertheless, the sample size reporting is inconsistent (n=476 vs. n=436) that need to be clarified. There are apparent typographical errors in certain tables (e.g. frequencies of the academic year). It would improve interpretation if effect sizes were reported in addition to p-values. Think about eliminating unnecessary repetition in the text and tables and providing clear justification for the covariates used in regression models. Additionally, to further improve methodological transparency, assumptions testing and handling missing data should be reported.

Author’s Reply: We thank you for this valuable comment. We have made the following changes in the Results and Statistical Analysis sections to address these issues. First, we have corrected the inconsistent sample size reporting throughout the manuscript and the final analytic sample is now consistently reported as n = 436. The typographical error in Table 1 related to the academic year frequency/percentage has also been corrected. To enhance the interpretation beyond the p-values, we have clearly presented the adjusted effect estimates from the multiple linear regression model as β coefficients with 95% confidence intervals in Table 3 and described the magnitude and direction of these associations in the Results section. We have added the effect size in the Table 2 for better interpretation. We further minimized overlap between the text and tables by discussing only main findings in the text, with detailed distributions in the tables, and we explained the reasons for covariate selection in the regression model, which was mainly based on theoretical relevance and previous literature, and kept household income because it was found to be statistically significantly associated with nomophobia in bivariate analysis at p < 0.05. To enhance methodological transparency, we also specified assumptions testing for parametric tests and regression models and clarified that missing data were minimal and handled using complete-case analysis. The changes were made in the revised manuscript as follows: Page 8, Lines 182; Page 12, Lines 209-211; Page 16, Line 228-235; Page 17, Lines 240-241, 244, 247-250; Page 18, Lines 254-255; Table 3.

(6) The thorough and properly referenced discussion successfully places the findings in both regional and global literature. But instead of providing a more thorough theoretical interpretation, it is repetitive and unduly descriptive, frequently restating the findings. Age and gender comparisons are erratic and require more precise synthesis. The conclusion could be shorter and more focused on prevention or intervention implications rather than generalizations. A greater focus should be placed on the limitations of sampling and the use of self-report.

Author’s Reply: Thanks for the valuable comment. We have revised the Discussion and Conclusion sections to reduce repetition, strengthen theoretical interpretation, improve synthesis of age and gender-related findings, and focus the conclusion more clearly on prevention and intervention implications. Firstly, we added a theoretical interpretation of the findings by linking higher nomophobia scores with behavioral dependence, cognitive salience, habitual smartphone checking, and anxiety related to disconnection. This was done to move the discussion beyond descriptive comparison with previous studies. We also added citations to support this theoretical interpretation. Secondly, we revised the discussion of age and gender for a more balanced synthesis. We made it clear that age-related findings are inconsistent between studies and should be interpreted with caution. For gender, we provided a more detailed explanation for the possible increased vulnerability of female students to nomophobia, including the greater use of smartphones for social networking, emotional communication, reassurance, maintenance of interpersonal relationships, and anxiety-related vulnerability. We also acknowledged that some studies

Attachment Submitted filename: Response_To_Reviewers_5.21.26.TC.docx

# Peer review: Decision Letter 1

**DOI:** 10.1371/journal.pone.0341524.r003

**Authors:** Syeda Humaida Hasan

21 Jun 2026

PLOS One

Dear Dr. Chowdhury,

Thank you for submitting your manuscript to PLOS ONE. After careful consideration, we feel that it has merit but does not fully meet PLOS ONE’s publication criteria as it currently stands. Therefore, we invite you to submit a revised version of the manuscript that addresses the points raised during the review process.

==============================

1. Please check the punctuation mark and any grammatical error.

'Phone use upon waking, and extensive engagement with social communication and media applications are associated with excessive smartphone use and the development of nomophobia; however, causality cannot be inferred due to the cross-sectional study design.”

The semicolon before “however” is missing and should be inserted.

2. Please avoid excessive multiple citations supporting a single statement in the introduction. In the sentence:

Relevant text  (“Although no notable gender differences were found in smartphone use, nomophobia scores between men and women showed remarkable deviation [14,17,18,20–24,26,30–32]”.)

The large number of references should be reduced and only the most relevant and representative studies should be cited to improve readability and avoid citation clutter.

3. Please clarify the exclusion criteria. The manuscript states that students who did not own a mobile device were excluded. However, it is unclear whether this refers to students without any mobile phone or specifically those without smartphones.

4.  The authors have not clearly stated whether the Nomophobia Questionnaire (NMP-Q) has been previously validated in the Bangladeshi population. The manuscript only mentions a pilot test. This issue should be addressed in the limitations section. The authors should also discuss the absence of formal psychometric validation (e.g., construct validity assessment) or provide additional validation analyses if available. [Relevant text:“Prior to the main study, the questionnaire underwent a pilot test among 60 participants to assess reliability using Cronbach’s alpha (α = 0.82).]

5. In line 207, please replace “higher nomophobia” with “higher nomophobia score” for clarity and scientific accuracy.

6.Several reported effect sizes (e.g., η² = 0.015) are marked. Please verify whether these have been correctly interpreted and whether they are statistically and practically meaningful. Ensure consistency in reporting and interpretation of effect sizes throughout the manuscript.

7. The statistical analysis section requires revision for consistency. The authors state that both multiple linear regression and logistic regression were used; however, the outcome variable (NMP-Q score) is continuous, and only multiple linear regression is presented in the results. No logistic regression analysis or corresponding results are provided in the manuscript. Therefore, the mention of logistic regression is misleading and should be removed or revised to accurately reflect the analysis performed. The authors should ensure that the statistical methods section is fully consistent with the reported analyses. Additionally, the description of regression assumption testing is overly detailed and should be streamlined to remove redundant wording.

==============================

Please submit your revised manuscript by Aug 05 2026 11:59PM. If you will need more time than this to complete your revisions, please reply to this message or contact the journal office at plosone@plos.org. When you're ready to submit your revision, log on to https://www.editorialmanager.com/pone/ and select the 'Submissions Needing Revision' folder to locate your manuscript file.

- A letter that responds to each point raised by the academic editor and reviewer(s). You should upload this letter as a separate file labeled 'Response to Reviewers'.
- A marked-up copy of your manuscript that highlights changes made to the original version. You should upload this as a separate file labeled 'Revised Manuscript with Track Changes'.
- An unmarked version of your revised paper without tracked changes. You should upload this as a separate file labeled 'Manuscript'.

If you would like to make changes to your financial disclosure, please include your updated statement in your cover letter. Guidelines for resubmitting your figure files are available below the reviewer comments at the end of this letter.

If applicable, we recommend that you deposit your laboratory protocols in protocols.io to enhance the reproducibility of your results. Protocols.io assigns your protocol its own identifier (DOI) so that it can be cited independently in the future. For instructions see: https://journals.plos.org/plosone/s/submission-guidelines#loc-laboratory-protocols. Additionally, PLOS ONE offers an option for publishing peer-reviewed Lab Protocol articles, which describe protocols hosted on protocols.io. Read more information on sharing protocols at https://plos.org/protocols?utm_medium=editorial-email&utm_source=authorletters&utm_campaign=protocols.

As the corresponding author, your ORCID iD is verified in the submission system and will appear in the published article. PLOS supports the use of ORCID, and we encourage all coauthors to register for an ORCID iD and use it as well. Please encourage your coauthors to verify their ORCID iD within the submission system before final acceptance, as unverified ORCID iDs will not appear in the published article. *Only*  the individual author can complete the verification step; PLOS staff *cannot*  verify ORCID iDs on behalf of authors.

We look forward to receiving your revised manuscript.

Kind regards,

Syeda Humaida Hasan, Diploma in Child Health, FCPS (Pediatrics)

Academic Editor

PLOS One

Journal Requirements:

If the reviewer comments include a recommendation to cite specific previously published works, please review and evaluate these publications to determine whether they are relevant and should be cited. There is no requirement to cite these works unless the editor has indicated otherwise.

Please review your reference list to ensure that it is complete and correct. If you have cited papers that have been retracted, please include the rationale for doing so in the manuscript text, or remove these references and replace them with relevant current references. Any changes to the reference list should be mentioned in the rebuttal letter that accompanies your revised manuscript. If you need to cite a retracted article, indicate the article’s retracted status in the References list and also include a citation and full reference for the retraction notice.

[Note: HTML markup is below. Please do not edit.]

Reviewers' comments:

Reviewer's Responses to Questions

**Comments to the Author**

Reviewer #1: All comments have been addressed

Reviewer #2: All comments have been addressed

**********

2. Is the manuscript technically sound, and do the data support the conclusions?

Reviewer #1: Yes

Reviewer #2: Yes

**********

3. Has the statistical analysis been performed appropriately and rigorously?

Reviewer #1: Yes

Reviewer #2: Yes

**********

4. Have the authors made all data underlying the findings in their manuscript fully available?

The PLOS Data policy

Reviewer #1: Yes

Reviewer #2: Yes

**********

5. Is the manuscript presented in an intelligible fashion and written in standard English?

Reviewer #1: Yes

Reviewer #2: Yes

**********

Reviewer #1: I thoroughly examined the updated manuscript and discovered that the authors had successfully incorporated the recommendations from the earlier review. It does not require further changes.

Reviewer #2: I would like to thank the authors for their meticulous efforts in thoroughly addressing all my concerns in this revision. The statistical rigor has been significantly improved by properly testing the regression assumptions and including the R² value. Furthermore, the distinction between medical and non-medical specializations is now clear, and the data presentation has been streamlined beautifully. The discussion section provides meaningful, well-reasoned socio-behavioral explanations for the findings. The manuscript now fully meets the high standards of PLOS ONE. I recommend it for final acceptance.

**********

what does this mean?). If published, this will include your full peer review and any attached files.

If you choose “no”, your identity will remain anonymous but your review may still be made public.

**Do you want your identity to be public for this peer review?**  For information about this choice, including consent withdrawal, please see our Privacy Policy

Reviewer #1: **Yes:**  Gyanesh Kumar Tiwari

Reviewer #2: **Yes:**  Mahmud Hamakareem Mahmud

**********

[NOTE: If reviewer comments were submitted as an attachment file, they will be attached to this email and accessible via the submission site. Please log into your account, locate the manuscript record, and check for the action link "View Attachments". If this link does not appear, there are no attachment files.]

To ensure your figures meet our technical requirements, please review our figure guidelines: https://journals.plos.org/plosone/s/figures

You may also use PLOS’s free figure tool, NAAS, to help you prepare publication quality figures: https://journals.plos.org/plosone/s/figures#loc-tools-for-figure-preparation.

NAAS will assess whether your figures meet our technical requirements by comparing each figure against our figure specifications.

# Author comment: Author response to Decision Letter 2

**DOI:** 10.1371/journal.pone.0341524.r004

28 Jul 2026

Response to Reviewer’s Comments

Journal Requirements:

Editorial Comments:

1. Please check the punctuation mark and any grammatical error.

'Phone use upon waking, and extensive engagement with social communication and media applications are associated with excessive smartphone use and the development of nomophobia; however, causality cannot be inferred due to the cross-sectional study design.”

The semicolon before “however” is missing and should be inserted.

Author’s Reply: We thank the academic editor for pointing this out. We have revised the sentence by inserting a semicolon before “however” and a comma after “however.” We also removed the unnecessary comma after “waking” and added “the” before “development” to improve grammatical accuracy and readability. This correction has been made in the conclusion section of the abstract. The revised manuscript has been modified as follows: Page 3, Lines 59, 60; Page 7, Lines 150-151 161, 163-164; Page 13, Lines 243.

2. Please avoid excessive multiple citations supporting a single statement in the introduction. In the sentence:

Relevant text (“Although no notable gender differences were found in smartphone use, nomophobia scores between men and women showed remarkable deviation [14,17,18,20–24,26,30–32]”.)

The large number of references should be reduced and only the most relevant and representative studies should be cited to improve readability and avoid citation clutter.

Author’s Reply: Thank you for this helpful suggestion. We agree that the previous sentence contained excessive citations supporting a single statement, which reduced readability. To address this, we have revised the sentence by breaking it into multiple sentences and retaining only the most relevant and representative references for each specific claim. The revised manuscript has been modified as follows: Page 4, Lines 90-94.

3. Please clarify the exclusion criteria. The manuscript states that students who did not own a mobile device were excluded. However, it is unclear whether this refers to students without any mobile phone or specifically those without smartphones.

Author’s Reply: Thank you for pointing out this ambiguity. We have clarified the exclusion criteria in the Methods section. The exclusion criterion referred to students who did not own any type of personal mobile phone, including button phones, multimedia/feature phones, or smartphones as well as any type of digital devices. The revised manuscript has been modified as follows. Page 6, Lines 132-136.

4. The authors have not clearly stated whether the Nomophobia Questionnaire (NMP-Q) has been previously validated in the Bangladeshi population. The manuscript only mentions a pilot test. This issue should be addressed in the limitations section. The authors should also discuss the absence of formal psychometric validation (e.g., construct validity assessment) or provide additional validation analyses if available. [Relevant text:“Prior to the main study, the questionnaire underwent a pilot test among 60 participants to assess reliability using Cronbach’s alpha (α = 0.82).]

Author’s Reply: Thank you for this important comment. We have revised the Methods section to clarify the use of the NMP-Q and to distinguish reliability assessment from formal psychometric validation. The questionnaire was pilot-tested in the present study and showed good internal consistency reliability, with a Cronbach’s alpha of 0.82. However, we acknowledge that no formal construct validity assessment, such as exploratory or confirmatory factor analysis, was conducted in our sample. We have therefore added this issue to the Limitations section and interpreted the findings accordingly. The revised manuscript has been modified as follows: Page 6, Lines 137-142, Page 23, Lines 391-399.

5. In line 207, please replace “higher nomophobia” with “higher nomophobia score” for clarity and scientific accuracy.

Author’s Reply: Thank you for this suggestion. We have replaced “higher nomophobia” with “higher nomophobia scores” to improve clarity and scientific accuracy. The revised manuscript has been modified as follows: Page 13, Line 219.

6.Several reported effect sizes (e.g., η² = 0.015) are marked. Please verify whether these have been correctly interpreted and whether they are statistically and practically meaningful. Ensure consistency in reporting and interpretation of effect sizes throughout the manuscript.

Author’s Reply: Thank you for this important comment. We have rechecked the reported effect sizes and revised the interpretation throughout the manuscript. We clarified that statistically significant findings with small η² values, such as η² = 0.015, indicate small practical effects despite statistical significance. We also added a table footnote describing the interpretation thresholds for Cohen’s d and η² to ensure consistency in reporting. Effect sizes are now interpreted cautiously and are not overstated as practically meaningful when their magnitude is small. The revised manuscript has been modified as follows. During rechecking, we identified an incorrectly reported η² value for ‘Via Mobile Data.’ Since η² values must range between 0 and 1, this value was recalculated and corrected in Table 2. The revised manuscript has been modified as follows: Page 13, Lines 225-233; Table 2; Page 17, Lines 239-241.

7. The statistical analysis section requires revision for consistency. The authors state that both multiple linear regression and logistic regression were used; however, the outcome variable (NMP-Q score) is continuous, and only multiple linear regression is presented in the results. No logistic regression analysis or corresponding results are provided in the manuscript. Therefore, the mention of logistic regression is misleading and should be removed or revised to accurately reflect the analysis performed. The authors should ensure that the statistical methods section is fully consistent with the reported analyses. Additionally, the description of regression assumption testing is overly detailed and should be streamlined to remove redundant wording.

Author’s Reply: Thank you for this important observation. We agree that the previous Statistical analysis section was inconsistent with the analyses reported in the Results section. Since the outcome variable was the continuous NMP-Q total score and only multiple linear regression was presented, we have removed the references to logistic regression. The Statistical analysis section has been revised to accurately describe the analyses performed, including t-tests, ANOVA, Pearson correlation, and multiple linear regression. We have also streamlined the description of regression assumption testing to avoid redundant wording. The revised manuscript has been modified as follows: Page 7, Lines 161, 163-165; Page 8, Lines 171-185.

Comments by Trisha Mae Tañedo Perez

1. Please include a legend for figure 1.

Author’s Reply: We thank the editor for this important observation. We have added a legend for figure 1. The revised manuscript has been modified as follows in the tracked change version: Page 13, Lines 238-242.

2. Please upload a copy of Figure 1 which you refer to in your text on page 12. Or if the figure is no longer to be included as part of the submission, please remove all reference to it within the text.

Author’s Reply: Thank you for this important suggestion. We have uploaded a copy of Figure 1 as follows: Figure “Figure_1.tiff” and Supporting Information “S2 File_5.21.26.tiff”.

6. Review Comments to the Author

Reviewer #1

I thoroughly examined the updated manuscript and discovered that the authors had successfully incorporated the recommendations from the earlier review. It does not require further changes.

Author’s Reply: We sincerely thank the reviewer for carefully re-evaluating the revised manuscript and for acknowledging that the previous recommendations have been satisfactorily addressed. We are grateful for the reviewer’s constructive comments, which helped improve the clarity, consistency, and overall quality of the manuscript.

Reviewer #2

I would like to thank the authors for their meticulous efforts in thoroughly addressing all my concerns in this revision. The statistical rigor has been significantly improved by properly testing the regression assumptions and including the R² value. Furthermore, the distinction between medical and non-medical specializations is now clear, and the data presentation has been streamlined beautifully. The discussion section provides meaningful, well-reasoned socio-behavioral explanations for the findings. The manuscript now fully meets the high standards of PLOS ONE. I recommend it for final acceptance.

Author’s Reply: We sincerely thank the reviewer for the careful re-evaluation of our revised manuscript and for the generous and encouraging feedback. We are grateful that the reviewer found the revisions satisfactory, particularly regarding the improved statistical reporting, clarification of academic specialization, streamlined data presentation, and strengthened discussion. The reviewer’s thoughtful comments throughout the review process were invaluable in improving the clarity, rigor, and overall quality of the manuscript. We deeply appreciate the recommendation for final acceptance.

Attachment Submitted filename: Response to Reviewers_7.28.26_TC.docx

# Editor report: Decision Letter 2

**DOI:** 10.1371/journal.pone.0341524.r005

**Authors:** Syeda Humaida Hasan

5 Aug 2026

PREVALENCE AND ASSOCIATED FACTORS OF NOMOPHOBIA AMONG THE MEDICAL AND UNIVERSITY STUDENTS OF BANGLADESH

PONE-D-26-01093R2

Dear Dr. Chowdhury,

We’re pleased to inform you that your manuscript has been judged scientifically suitable for publication and will be formally accepted for publication once it meets all outstanding technical requirements.

Within one week, you’ll receive an e-mail detailing the required amendments. When these have been addressed, you’ll receive a formal acceptance letter and your manuscript will be scheduled for publication.

An invoice will be generated when your article is formally accepted. Please note, if your institution has a publishing partnership with PLOS and your article meets the relevant criteria, all or part of your publication costs will be covered. Please make sure your user information is up-to-date by logging into Editorial Manager at Editorial Manager® and clicking the ‘Update My Information' link at the top of the page. For questions related to billing, please contact billing support.

If your institution or institutions have a press office, please notify them about your upcoming paper to help maximize its impact. If they’ll be preparing press materials, please inform our press team as soon as possible -- no later than 48 hours after receiving the formal acceptance. Your manuscript will remain under strict press embargo until 2 pm Eastern Time on the date of publication. For more information, please contact onepress@plos.org.

Kind regards,

Syeda Humaida Hasan, Diploma in Child Health, FCPS (Pediatrics)

Academic Editor

PLOS One

Additional Editor Comments (optional):

Multiple citations for a single statement are also repeated in other sentences. Please check all references carefully to ensure they are cited appropriately and presented in the correct chronological order. Additionally, Reference 31 is missing.

Reviewers' comments:

# Editor report: Acceptance letter

**DOI:** 10.1371/journal.pone.0341524.r006

**Authors:** Syeda Humaida Hasan

PONE-D-26-01093R2

PLOS One

Dear Dr. Chowdhury,

I'm pleased to inform you that your manuscript has been deemed suitable for publication in PLOS One. Congratulations! Your manuscript is now being handed over to our production team.

At this stage, our production department will prepare your paper for publication. This includes ensuring the following:

* All references, tables, and figures are properly cited

* All relevant supporting information is included in the manuscript submission,

* There are no issues that prevent the paper from being properly typeset

You will receive further instructions from the production team, including instructions on how to review your proof when it is ready. Please keep in mind that we are working through a large volume of accepted articles, so please give us a few days to review your paper and let you know the next and final steps.

Lastly, if your institution or institutions have a press office, please let them know about your upcoming paper now to help maximize its impact. If they'll be preparing press materials, please inform our press team within the next 48 hours. Your manuscript will remain under strict press embargo until 2 pm Eastern Time on the date of publication. For more information, please contact onepress@plos.org.

You will receive an invoice from PLOS for your publication fee after your manuscript has reached the completed accept phase. If you receive an email requesting payment before acceptance or for any other service, this may be a phishing scheme. Learn how to identify phishing emails and protect your accounts at https://explore.plos.org/phishing.

If we can help with anything else, please email us at customercare@plos.org.

Thank you for submitting your work to PLOS One and supporting open access.

Kind regards,

PLOS One Editorial Office Staff

on behalf of

Dr. Syeda Humaida Hasan

Academic Editor

PLOS One
